# Original art paintings are chosen over their “color-rotated” versions because of changed color contrast

**DOI:** 10.1177/03010066251345994

**Published:** 2025-06-16

**Authors:** Bruno Laeng, Morten Øvervoll, Ece Aybike Ala-Pettersen

**Affiliations:** Department of Psychology, 6305 University of Oslo, Oslo, Norway; RITMO Centre for Interdisciplinary Studies in Rhythm, Time and Motion, 6305 University of Oslo, Norway; Department of Psychology, The Arctic University of Norway, Tromsø, Norway; Department of Psychology, University of Oslo, Oslo, Norway

**Keywords:** esthetics, liking, preference, arousal, forced choice, color, paintings, color contrast, saliency, color CIELAB, color rotation

## Abstract

Rotating colors (digitally within CIELAB color space) of an artistic painting is thought to keep constant all aspects of the painting except the hues. When observers are asked to select the preferred image among color-rotated images the “original” version of the artwork is typically selected, while the hue transformed images are rejected. We hypothesized that color contrast may be reduced after such digital rotations, which was supported by feature analyses. We also found that when the original painting or rotations were viewed individually, they did not differ in both hedonic ratings and pupil responses, though observers selected the original paintings in a forced-choice test. Hence, we generated versions of the paintings where color contrast was either enhanced or reduced and forced-choice experiments (online or in the lab) confirmed that the higher color contrast image within a pair was preferred (regardless of whether the image was an original painting or not). Eye tracking revealed that images with relatively higher contrast captured attention. We conclude that previous reports of a preference for the original artworks may have reflected reductions in color contrast of the color-rotated alternatives. These findings point to color contrast as a potential esthetic primitive feature but at the same time cast some doubts on relying exclusively on the results of forced choice tests for revealing genuine esthetic preferences.

## Introduction

In 2022, major newspapers and magazines (e.g., *The Guardian,* article by Jacobs; *The New Yorker,* article by Gopnik) published the news that an art piece by Piet Mondrian had been hanging on the wall of the museum for 75 years in the erroneous, upside-down, orientation. This real episode and similar others (e.g., two Rothko's paintings shown rotated 90° for 9 years at the Tate in London) illustrate the possibility that a work of art may be just as appreciated when displayed in a manner radically different from that originally intended by the artists, possibly because—as in the present case of a spatial rotation—its features are harmonized in a manner that remains resistant to the change. An article about the same incident, published in the *New Statesman* (article by Cody)*,* explicitly carried the title “Mondrian's upside-down masterpiece is a reminder that art is completely subjective.” In psychological research in the laboratory, Johnson et al. (2010) showed images of real Mondrian paintings on a computer screen to naïve viewers, either in the original orientation or through different orientations (in steps of 45°), and found that, based on their subjective reports or ratings, all orientations were equally liked, though—unknown to them—their eye pupils (measured with an eye tracker) showed maximal dilations to the original orientation of each specific “Mondrian.”

The extent in which the originally intended presentation of a work of art may be essential to its meaning or appreciation remains a topic of discussion. It is likely that many other dimensions of an artwork besides its position in space may be either resistant to change or flexible. Color is certainly a prominent feature of much art and design and it seems plausible to think that “many great works of art would not be as impressive in grey scales” (Albers et al., 2020, p. 109). However, when considering sculpture history, many are today surprised to learn that the white marble statues of ancient Rome or Greece were systematically painted over in various colors (Hart, 2018). In fact, both artists and philosophers (e.g., Leonardo, Hegel, and Winckelmann) have celebrated for centuries the monochrome aspect of classical, Greco-Roman, marble sculptures without knowing that the original public (or artists) may have not liked them that way.

Focusing the discussion on paintings, the specific colors chosen by artists for paintings could be subjective or not crucial in most cases, so that either removing or exchanging the original colors may lead to change in preference for some viewers. Indeed, Braun and Doerschner (2019) allowed participants to adjust the color of a single element in 24 paintings and found that almost never they chose the same colors as the artists. When the same participants were asked to state their preference in a test where the original and the participant's version were shown together, they liked their own color choices about as much as the original art pieces.

**Figure 1.**
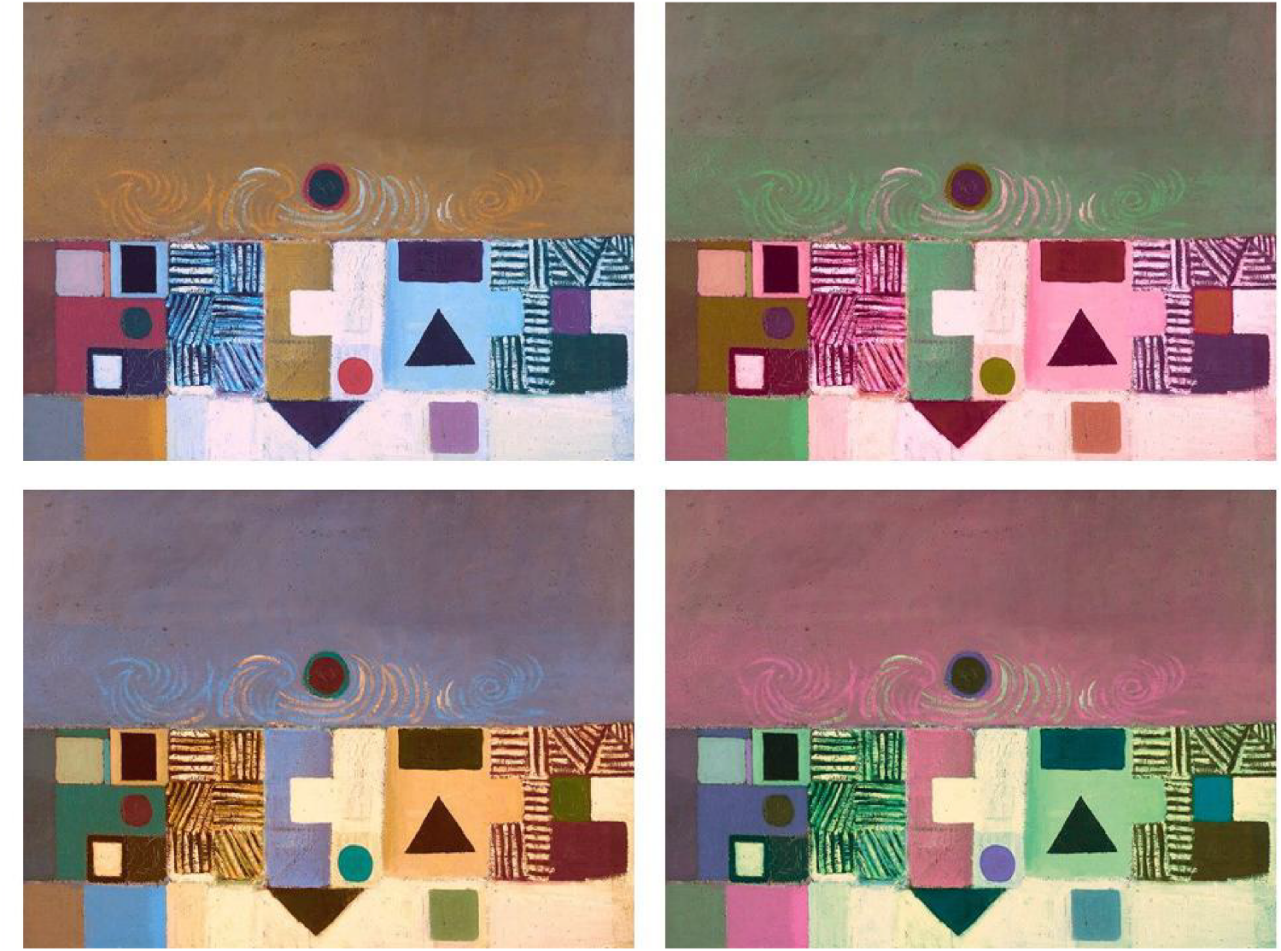
Painting by Victor Pasmore (1950) from WikiArt (fair use). Top left: original painting; clockwise: L* axis angle = 0°; hue-rotated: angles = 90°, 270°, 180°.

However, several recent psychological studies suggest that viewers, when given a chance to change the colors in real work of arts, though these are unknown to them, converge toward color combinations that are impressively like those of the original paintings (e.g., Nakauchi & Tamura, 2022; Nascimento et al., 2017). In these studies, taking advantage of a computerized set-up, the “color volume” of each painting could be “rotated” rigidly around the mean of the opponent color axes in CIELAB space, a technique that is meant to keep constant lightness and saturation while systematically changing the hues in the images. Note that these rotations around the means can maintain the relative distances of color hues and the relative luminance of each color patch, while changing the overall appearance of the hues in the artwork. Remarkably, these studies (Nakauchi et al., 2022; Nakauchi & Tamura, 2022; Nascimento et al., 2021) have converged in showing that, when groups of participants viewed the original painting and three other equally spaced rotations (e.g., as in Figure 1), and were asked to select one version as “the best” out of four, participants preferred on average the original painting. In this forced-choice procedure, the observer is led to focus on the chromatic colors in the images, since these are the only visible difference between the four images while other properties (e.g., luminance, spatial structure) remains unchanged. Note, however, that some “paint colors” are unaffected by the rotation procedure, like achromatic lines and patches of black or white paint.

Even more remarkable, some of the studies also provided evidence that the specific spatial compositions of the original colors were not necessary for obtaining the superiority in preference for the original colors. In fact, parsing digitally the canvases of different paintings in small bits and then randomly arranging them did not eliminate a preference for the original colors’ gamut (Nascimento et al., 2021). Also surprisingly, rearranging these bits from many different paintings in novel “patchworks” still led to a preference for the image made with original paintings’ colors than those from any of the hue-rotated versions, which led some of the researchers to conclude that something about the “color distribution statistics” of the original artworks is crucial for yielding the original colors’ superiority over those of the hue-rotated versions (Nakauchi et al., 2022). An initial account (Nascimento et al., 2017) proposed that artists might tend to apply colors over the canvas that optimally satisfy the sense of color harmony of other viewers, including those of art novices. However, this account runs into a problem with subsequent evidence that, parsing the original images and scrambling its parts (Albers et al., 2020) did not change the likelihood that the original gamut of colors was preferred. In fact, for color harmony, it should be important what are the colors that are bordering one another (Brenner et al., 2003; Conway, 2012).

In the present study, we consider the possibility that, despite rotating rigidly the hues around the mean of the opponent color axes in CIELAB is meant to leave undisturbed all the other features of a painting (e.g., saturation), it is possible that some other feature/s are weakened by the manipulation, leading the original to stand out among the less salient, transformed, versions. Although rotations around the mean of the opponent color axes may preserve features like lightness and saturation within perceptual thresholds, other visual changes may either affect the esthetic value of the composition across variations or generate an attentional bias when one is required (forced) to choose among the original and a less salient alternative.

## Experiment 1: Analyses of Contrast Within Two Color Spaces After Color Gamut Rotations

A plausible candidate feature that may be weakened by the hue-rotation procedure is the perceived contrast among colors. Note that *color contrast* is not the same as *luminance contrast*, which appears to be well controlled by the hue-rotation procedure used in all previous studies as well as the present one. To provide an example of color contrast, it is known that red or blue appear brighter than equiluminant yellow and green stimuli (a phenomenon called the Helmholtz-Kohlrausch effect; Corney et al., 2009). Also, blue evokes stronger pictorial glare effects than equiluminant colors (Suzuki et al., 2019). As an example of how these perceptual differences can affect behavior, when color contrast is high, normal individuals can read as rapidly as with high luminance contrast (e.g., Legge et al., 1990).

In physiological terms, to understand the perceptual effects of color gamut rotation, it is important to consider the relative stimulations of L-, M-, and S-wavelength cones, as well as the responses of opponent color cells. When rotating the opponent color axes a* and b* around the mean, while keeping lightness (L*) constant, the perceived magnitude of a rotating color will not remain stable over rotation, and the relationship between colors will change (e.g., perceptually opponent colors will not remain opponents for all rotations). In the brain, the chromatic channels are recombined in processing to form novel representations of color (e.g., Goddard et al., 2010). Thus, if we consider the multistage color model of De Valois and De Valois (1993) to estimate the responses of color-sensitive cortical cells, such a model translates an image into L, M, and S cone responses feeding into opponent retinal ganglion cells and LGN cells and produces cortical red-green and blue-yellow color responses. When colors are rotated as described in most of the literature (i.e., rotation around the mean color of the image, rotating a* and b*, while keeping L* constant), the color magnitude in CIELAB space varies systematically in principle, so that the relative distance between all colors remains constant over rotation. However, this is not necessarily the case in physiological color space.

**Figure 2.**
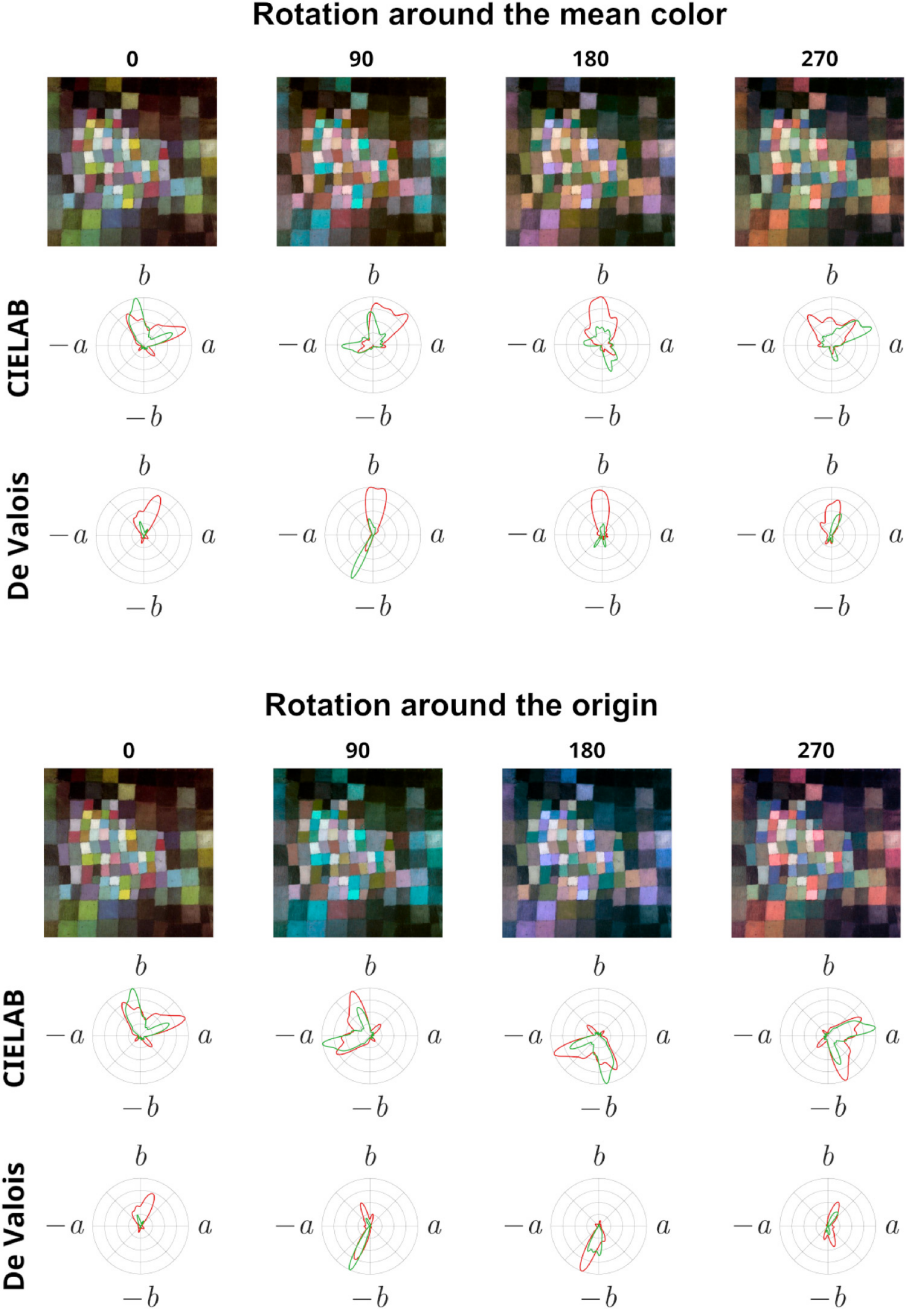
An original painting (0°) and the resulting images after three equally spaced gamut rotations (90°, 180°, and 270°) in CIELAB space around the mean color (top) or the origin (bottom). The polar plots, for CIELAB space and physiological (De Valois) color space, show the proportion of pixels within each hue (red line) and the average saturation of the pixels (green line), both normalized to the maximum value for all four versions of the painting.

As Figure 2 illustrates, three equally spaced gamut rotations of an original painting around the mean color or the origin in CIELAB color space—as typically done in several previous studies—affect the color distributions differently. For rotations around the mean color, the distance in CIELAB space between any two pixels, and between all pixels and the mean color, remains constant across gamut rotations, but the difference in hue and saturation between pixels does not remain constant (see Figure 2, top). For rotations around the origin, on the other hand, the difference in hue and saturation between pixels remains constant, and the distributions are merely shifted by the degree of rotation (see Figure 2, bottom). Rotation around the origin should therefore ensure a stable perceptual relationship between colors across rotations, but this presupposes a correspondence between CIELAB and perceptual color spaces. However, as also shown in Figure 2, the distributions of hue and saturation in physiological color space differ from the distributions in CIELAB color space, both for rotations around the mean color and the origin, suggesting that color gamut rotations in CIELAB color space change the perceived relationship between colors.

**Figure 3.**
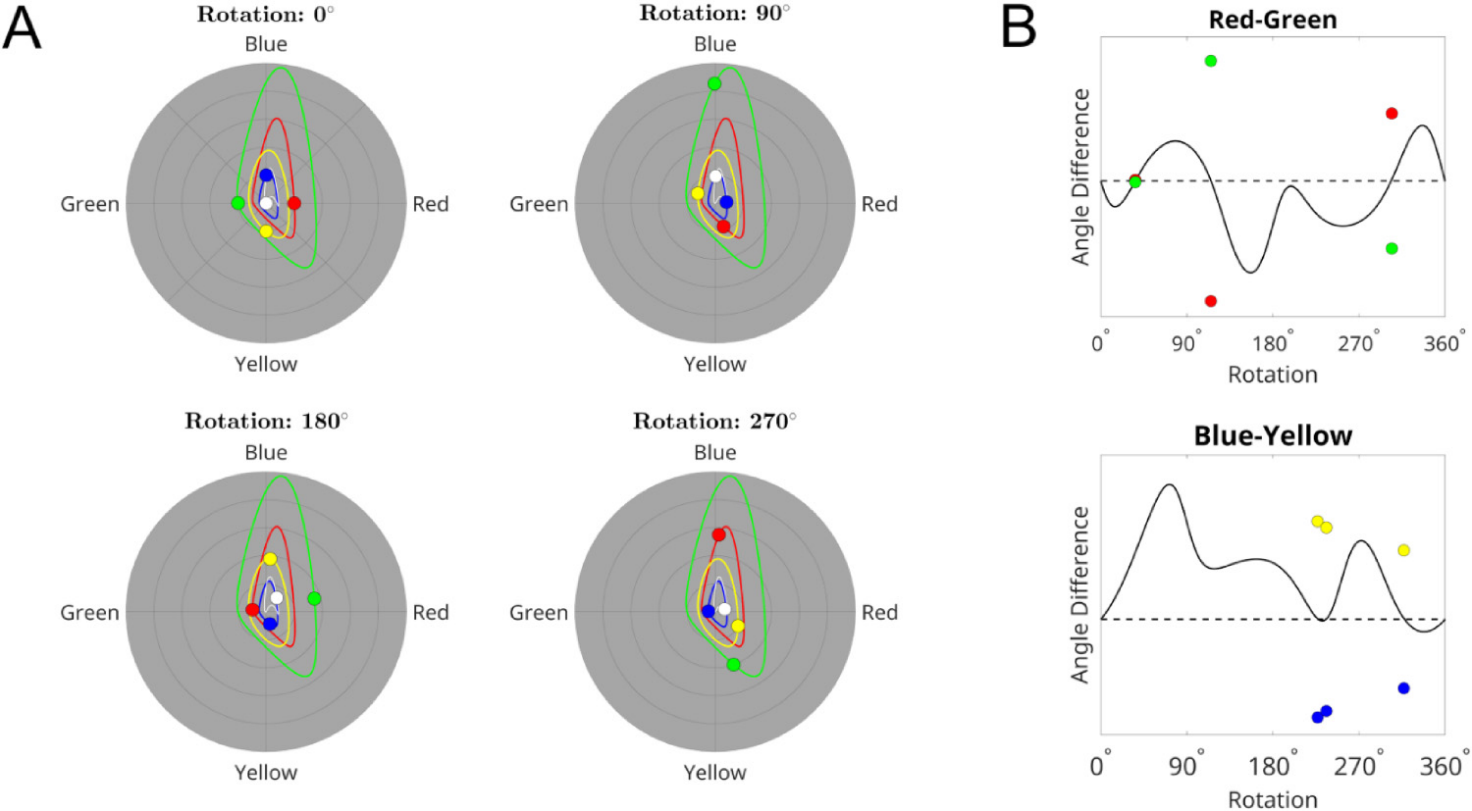
Representation of colors in physiological color space after rotating colors around the mean color in CIELAB color space. A. The color disks represent the position in physiological color space for two opponent color pairs (red-green and blue-yellow) that are matched in magnitude (top left panel), and are rotated 90°, 180°, and 270° clockwise around the mean color in CIELAB space. The white disk represents the average of the color responses. The curves represent the trajectories for the original colors (top left panel) for a continuous rotation around the mean in CIELAB space. B. The curves show the difference in angle in physiological color space between the opponent pairs in A, for a continuous rotation of the four colors around the mean in CIELAB color space. The dotted reference lines indicate a difference of 180° (opponency). For the points where the curves cross the line, the color disks show the relative magnitude of the pairs (highest/lowest magnitude is placed above/below the line).

Thus, it is plausible that different aspects of paintings become perceptually less salient as the CIELAB color gamut is rotated. As Figure 3A illustrates, the perceived magnitude of the colors may change differently in physiological color space after rotations that in CIELAB space. Both of the originally opponent color pairs remain opponent for only three points over all possible rotations (Figure 3B), and for these points the magnitude of the colors are not matched, although they are nearly matched for the first red-green point. So, for these four colors, there are no rotations where the colors remain opponent and matched in magnitude (opponent pairs with other hues or magnitudes will vary differently). While the color magnitude in CIELAB space varies predictably over rotation, where the distance between colors remains constant over rotations, this is not the case in physiological color space, where the distance between colors varies over rotation. Thus, the original relationship between colors might not remain stable in physiological color space after rotations in CIELAB space.

Based on the effects modeled in Figure 3, we consider that for the primary four colors together, there may be no rotations other than the original where the colors remain opponents and will be matched in strength. A possible consequence of this is that color contrast could be highest for the original and lower for all rotations. Such changes might not only affect the opponent colors, but they could be widespread over the whole color gamut, because hue and saturation might not vary smoothly and systematically over rotation in physiological space as they do in the CIELAB space.

It seems likely that in the visual arts, some painters may intentionally seek to maximize color contrast; an example is provided by previous analyses of the paintings by van Gogh that showed a frequent use of complementary colors (Berezhnoy et al., 2007), that is opposite colors in terms of hues, that strongly enhance the chromatic properties of colors and contours of objects in his paintings (Li & Chen, 2009; Mamassian, 2008). Thus, we surmise that rotations in CIELAB color gamut would particularly reduce the perceived color contrast compared to the original paintings. Moreover, a recent computational analysis employing a deep convolutional neural network to identify the distinct visual features in art paintings as well as photographs supports the view that a low-level feature like (luminance) contrast can predict the viewers’ esthetic preferences (Iigaya et al., 2021; Reber et al., 2004). Indeed, contrast (NB: in luminance) appear to be one of the common factors (together with symmetry, complexity, proportion, contour, brightness) underlying esthetic preferences (Juricevic et al., 2010; McAdams et al., 2023), often shared across cultures (Che et al., 2018). Luminance contrast can also affect preference in other domains, like facial attractiveness from photos (Russell, 2003). Unsurprisingly, Krentz and Earl (2013) showed that reducing luminance contrast in paintings and then forcing adults or children to choose one of the two in a pair, leads to the originals being chosen (or looked at) more frequently than the degraded versions.

We believe that color contrast is a feature that is likely to change dramatically when transforming hues, given the expected compression effects of rotations within the color opponency channels. Moreover, as discussed by Altmann et al. (2021), if gamut rotations reduce the number of perceived color categories and, consequently, their perceived color contrasts, then participants may tend to choose those paintings that look “richer” in colors or appear to have more color variance or complexity. Therefore, we begin by presenting an analysis where we computed color contrast within two color spaces: The CIELAB, as in all previous studies, as well as in an alternative model of physiological color space, that we based on the multistage color model of De Valois and De Valois (1993). Our predictions are that applying color rotations will lead to significant physical changes in the above measure and, likely, within both the “De Valois” color space than the CIELAB spaces, though with different strength.

### Method

*Stimuli.* We analyzed 22 original abstract art paintings obtained from Yuma Taniyama and Shigeki Nakauchi (Toyohashi University of Technology in Japan), originally selected from “fair use” images in *WikiArt* (https://www.wikiart.org/, search key: genre Abstract paintings). This selection of paintings covers an art period ranging from 1913 to 2007, with the majority being created in the second half of the 1900s. A few of the selected paintings were by well-known artists (e.g., Fernand Leger, Joan Mitchell). Paintings’ sizes were normalized by bilinear interpolation, setting the smallest axis to 512 pixels while preserving the aspect ratio. The color gamuts of the paintings were rotated following the procedure described by Nascimento et al. (2017), where the L* plane of the CIELAB color space was kept constant while the a* b* coordinate for each pixel in the image was rotated around the mean color of the image. All images were rotated from −180° to 180° (−π to π radians, in steps of 0.01π), with the original image as a starting point for each rotation. Points falling outside the sRGB gamut after rotation were set to the surface boundary of the sRGB gamut projected in CIELAB space, by reducing saturation while keeping the hue angle. All analyses were performed in MATLAB R2022a.

#### Physiological or “De Valois” Color Space

The multistage color model of De Valois and De Valois (1993) were implemented to simulate responses of “red-green” and “yellow-blue” sensitive neurons. In the first stage of the model, cone responses were calculated from sRGB values (range 0 to 1), according to Equation 1. The original model uses Smith and Pokorny’s (1975) cone fundamentals, but because their S-cone fundamental has been shown to fit poorly with measured spectral sensitivity (Stockman & Sharpe, 1998), we used Stockman and Sharpe’s (2000) 2° cone fundamentals. The transformation matrix is derived from the RGB phosphor emission curves of a gamma-corrected Sony F520 Trinitron monitor (Westland, 2021).

(1) $$\left(\begin{array}{c} L\\ M\\ S \end{array}\right)=\left(\begin{array}{ccc} 0.04579 & 0.07314 & 0.01291\\ 0.01620 & 0.07527 & 0.01834\\ 0.00164 & 0.00547 & 0.06786 \end{array}\right)\left(\begin{array}{c} R\\ G\\ B \end{array}\right)$$

Cone responses were then normalized by converting to cone contrast (Cole & Hine, 1992; De Valois et al., 1997), defined here as relative change in response from a middle gray field (Equation 2, where *L*_gr_, *M*_gr_ and *S*_gr_ are cone responses to the RGB triplet [0.5, 0.5, 0.5].)

(2) $$\begin{array}{rl} & L^{\prime }=(L-L_{gr})/L_{gr}\\ & M^{\prime }=(M-M_{gr})/M_{gr}\\ & S^{\prime }=(S-S_{gr})/S_{gr} \end{array}$$

In the second stage, cone opponency was calculated using the indiscriminate version of the model (Equation 3). The center cone is given a weight of 16 and is assumed to be affected by 16 surrounding cones of all types in the ratio 10L:5M:1S, which is subtracted from the center.

(3) $$\begin{array}{ccccccc} L_{0} & \;= & 6L^{\prime } & - & 5M^{\prime } & - & S^{\prime }\\ M_{o} & = & â{ˆ}^{\prime }10L^{\prime } & + & 11M^{\prime } & - & S^{\prime }\\ S_{o} & = & â{ˆ}^{\prime }10L^{\prime } & - & 5M^{\prime } & + & 15S^{\prime } \end{array}$$

In the third stage, “red-green” (*RG*) and “yellow-blue” (*YB*) opponent color channels were calculated according to Equation 4, by subtracting M_o_ from L_o_ for the “red-green” channel, and L_o_ from M_o_ for the “yellow-blue” channel and modulating both by S_o_ which is doubled in weight from the second stage.

(4) $$\begin{array}{ccccccc} RG & = & 10L_{o} & - & 5M_{o} & + & 2S_{o}\\ YB & = & -10L_{o} & + & 5M_{o} & + & 2S_{o} \end{array}$$

#### Color Contrast

The estimation of color contrast is based on the complex representation of the opponent color axes (a* + ib* or *RG* + *iYB*). It resembles RMS contrast, as it is an estimate of the mean difference from the mean. However, we use distances from the mean in complex space instead of squared differences from the mean grayscale value (RMS contrast). Contrast was computed according to Equation 5, where *ab* is the complex representation of the opponent color axes for all pixels. Here, the distance for each pixel is weighted by the pixel's magnitude, so that a highly saturated pixel with a given distance from the mean will have a greater impact than a desaturated pixel with the same distance from the mean. Before computing contrast, *ab* was normalized by dividing with the mean magnitude (i.e., setting the mean magnitude of the image to 1).

(5) $$C=\frac{1}{MN}\sum_{i=1}^{M}\sum_{\;j=1}^{N}\left|ab_{ij}(ab_{ij}-\bar{ab}\right)|$$

### Results

**Figure 4.**
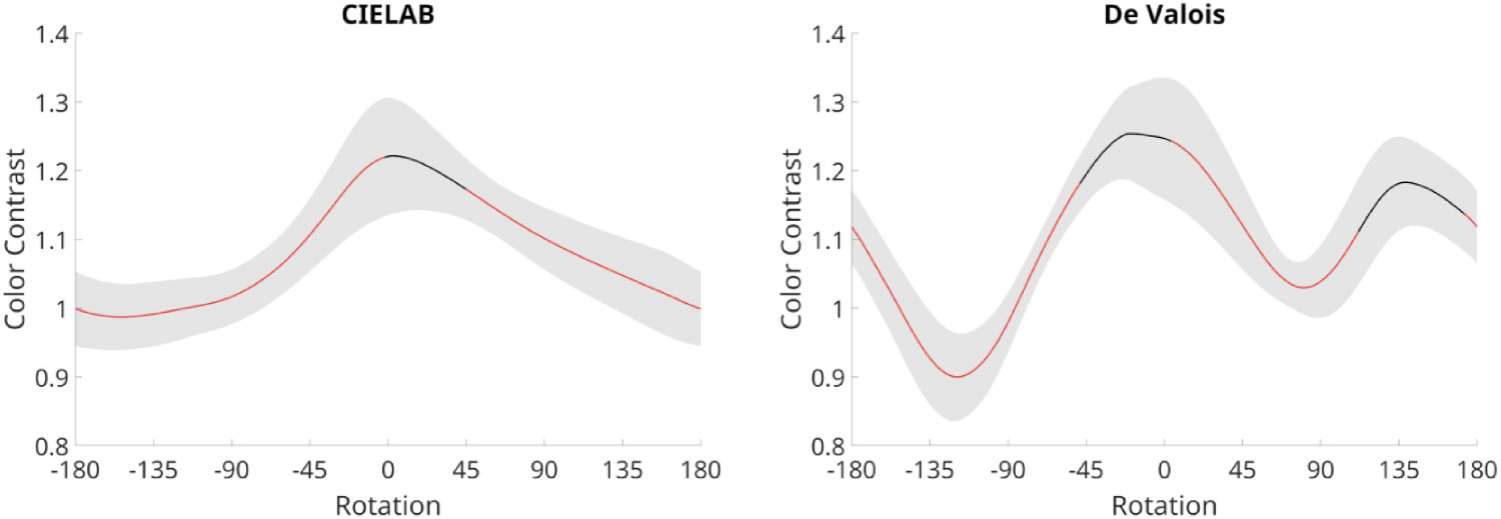
Graphs illustrating changes in color contrast within CIELAB (left column) and De Valois (right column) spaces. Red portions of the lines indicate significant differences from the original image. The gray shadings represent 95% within-group confidence intervals.

We used nonparametric permutation testing (providing strong FWER control; cf., Groppe et al., 2011) with *t*-tests and threshold free cluster enhancement (Mensen & Khatami, 2013; Pernet et al., 2015) and 10,000 permutations (alpha level = .05) to compare all rotated versions against the original painting. Figure 4 shows the averaged continuous changes in color contrast within CIELAB and De Valois spaces for all paintings. Compared to the original painting, color contrast decreased significantly after rotation of the color gamut, both in CIELAB and De Valois space.

Two separate repeated-measure ANOVAs were run on the obtained contrast values for each painting, as the random variable, and Angle (0°, 90°, 180°, and 270°) as the within-factor. The analysis of color contrast within CIELAB space showed a significant effect of Angle (*F*(3, 63) = 8.36, *p* < .0001; η² = 0.285).

**Figure 5.**
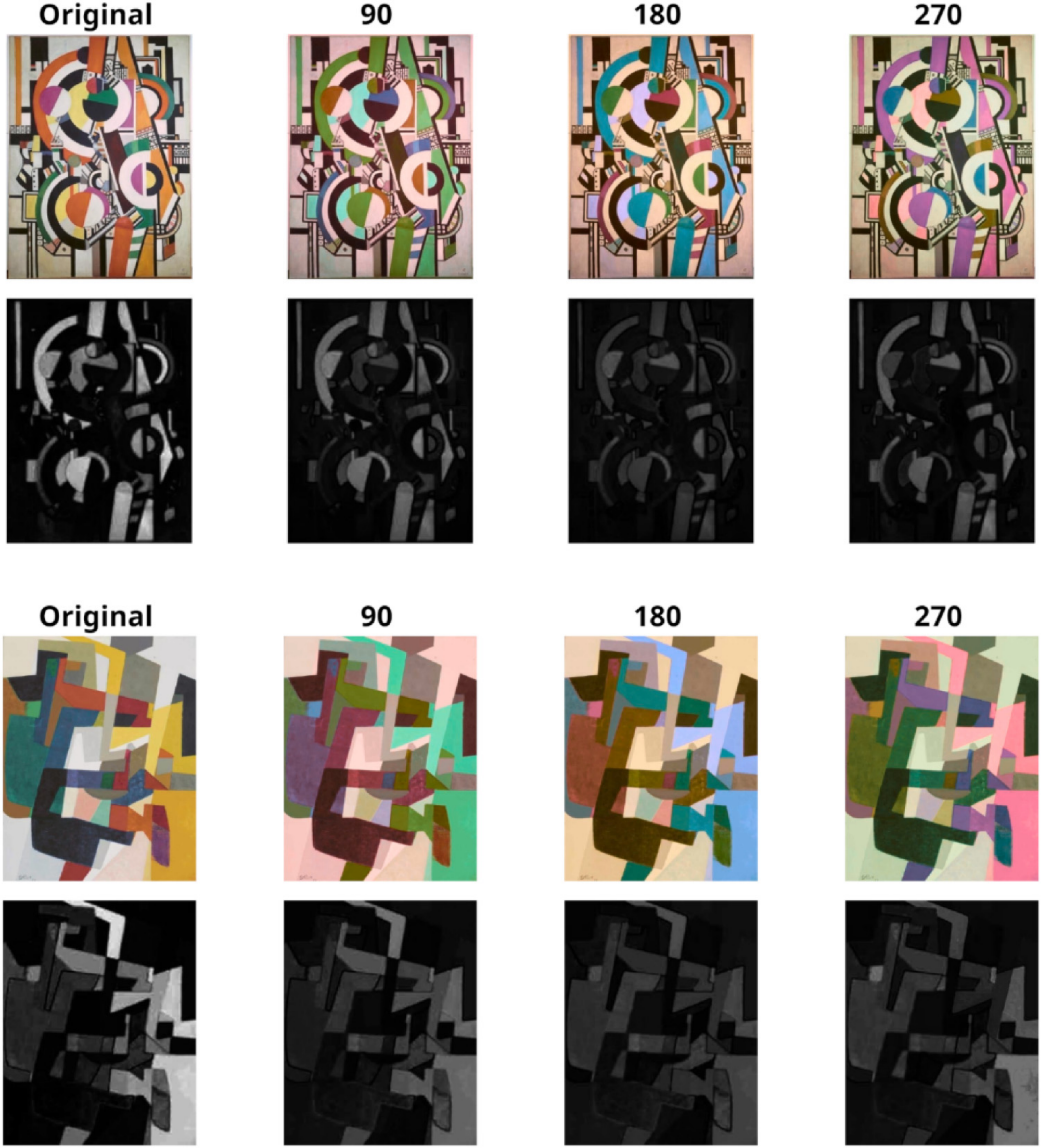
Visualization of color contrasts within De Valois space for two of the paintings after 0° (original), 90°, 180°, and 270° rotation around the mean color in CIELAB color space. The grayscale images show each pixel’s distance from the mean color.

Contrast was highest on average for the original painting (M = 1.221; SE = .080) than the rotations (Angle 90°: M = 1.101; SE = .054; Angle 180°: M = 1.001; SE = .046; Angle 270°: M = 1.017; SE = .049). Analogously, the analysis of color contrast within De Valois space, showed a significant effect of Angle, *F*(3, 63) = 10.18, *p* < .0001; η² = 0.327. Contrast was highest on average for the original painting (M = 1.247; SE = .089) than the rotations (Angle 90°: M = 1.039; SE = .078; Angle 180°: M = 1.116; SE = .059; Angle 270°: M = 0.98; SE = .063). To visualize reductions in color contrast introduced by each of the three angles of rotation away from the original, we show in Figure 5 two examples of paintings (in color) and below each angle of rotation (in grayscale), the corresponding color contrast levels (i.e., each pixel's distance from the mean, according to Equation 5 before the average distance is calculated).

A linear regression between the color contrast obtained for all paintings in the CIELAB and De Valois spaces, revealed that the two measurements were very highly related, *r* = .974 (when using originals) and *r* = .903 (when adding the rotated versions).

### Discussion

Our computations of color contrast showed significant reductions with rotations away from the original painting. Contrast consisted of a global measure of the difference between the colors in the image (in opponent color space, a* and b*, or RG and YB). For example, a painting consisting of several areas with opponent colors will have a higher color contrast than a painting consisting of several areas with more similar hues. When contrast is high, the visibility of the image is also high, but based on chromatic differences only (not in luminance). We found significant reductions after rotations and in both color spaces (Figure 4), though they tended to yield larger effect sizes in De Valois space than CIELAB. As expected, the original paintings showed the highest levels of color contrast. Interestingly, both color spaces yielded highly related estimates of color contrast for all paintings.

It is likely that color contrast is not the only sensory feature that may be affected by the hue rotation in CIELAB space. As mentioned in previous studies (e.g., Nascimento et al., 2017, 2021), the hue rotation does cause a variable gamut compression due to the limited volume of the colors reproducible by monitors. Despite these changes in saturation are estimated to be rather small and possibly negligible, since they amount to near-threshold values for detecting a chromatic change (Schiller & Gegenfurtner, 2016), it cannot be excluded that even nearly visible changes in saturation could influence a forced choice, also because color saturation is one of the features that is known to affect color preference (e.g., Camgöz et al., 2002; McAdams et al., 2023). Furthermore, Altmann et al. (2021) found that their participants judged 95% of all the original paintings used in their study as more colorful than their manipulated counterparts. They surmised that presenting the images on LCD monitors causes a disproportionate compression for some colors and it remains theoretically possible that CIELAB color values risk to be out-of-gamut after conversion on sRGB displays. Another concern, that we specifically addressed, is that there seems to be a clear limitation of the CIELAB space in representing the “perceived” saturation values; hence, there is a need for developing color spaces that better approximate the physiological and perceptual responses of human color vision.

## Experiment 2: Hedonic Ratings of the Color-Rotated Paintings and Pupil Responses

If the preference exposed in forced-choice tests with hue-rotated paintings reflects esthetic preferences for the original paintings, it is straightforward to expect that the originals should also receive the highest esthetic ratings when participants are requested to assign a hedonic value on a scale. Indeed, subjective ratings and forced choices can be considered alternative methods for probing esthetic preferences, each having different strengths and weaknesses. Both forced choices and subjective ratings of pleasantness (e.g., Lyssenko et al., 2016) have been widely used, but rarely together, as valid methods for investigating people's judgments of artworks. In fact, ratings provide a form of ipsative judgment that has some advantages over forced choices.

Forced choices have the strength of being able to single out a winner in a consistent manner even in cases in which the subjective hedonic values differ only slightly and, plausibly, this has been the reason of nearly all studies on the effects of color rotations relying exclusively on this measure. The weakness of the forced-choice method, which is typically not discussed in studies on color rotations, is that it may provide results even when, in esthetic terms, the viewed stimuli are indifferent or in truth all disliked but in different degrees. In fact, given that the forced choice procedures only provide a relative winner among a set of items, they cannot reveal an explicit hedonic value, that is the liking or disliking of each stimulus, and to what degrees on an interval scale (Lim, 2011). Note that there is also a qualitative, contextual difference between the two methods for measuring hedonic judgements: The forced-choice method, by definition, relies on a more relative type of judgment, making one choice among at least a pair of visible items, whereas in active rating*,* only one image is typically shown at any given time. However, such a contextual difference mainly yields complementary paths for capturing esthetic appeal; the subjective scaling obtained with ratings is capable to probe hedonic state in a direct manner and when considering the artwork by itself, without making relative comparisons, as it is often the case in everyday situations. Moreover, the procedure of assigning a numerical value that indicates value level, is widespread in ordinary human affairs (like prizes, critics of restaurants, some sports, as well as a good deal of the educational evaluation system), mainly because it explicitly allows specifying the valence of a judgment, as well as allowing the choice of a zero point or the possibility that many judgments remain neutral (Dhar & Simonson, 2003; O’Mahony & Wichchukit, 2017). Hence, ratings can identify occurrences in which observers do not specifically find esthetic appeal for any of the items and therefore select—by force—the least disliked. For this reason, ratings open the possibility of a study to fail to give results, while forced choices always yield results. This may be taken as a weakness, but it could be seen instead as a strength, since a lack of convergence among different hedonic estimates may signal the presence of other biases affecting choices, as we will discuss in the last section of the general discussion.

Given the tradeoff of benefits and costs with the two methods, collecting both hedonic ratings and forced choices, or seeking converging evidence, seems the best strategy for investigating genuine esthetic responses. Thus, in the following experiment, we used odd-parity seven-step Likert scales for hedonic ratings, thereby allowing the use of a middle option (the “4” rating) to express neutrality or indifference about the presented items as well as a full range of negative judgements. Consequently, we requested our same participants to participate also in a 4-forced-choice test, so that we could compare results with both methods and the same participants. Note that the present study may be the first to use both methods together instead of only forced choices (e.g., Albers et al., 2020) or only ratings (Altmann et al., 2021).

An important addition of this study is that we assess whether preferences are reflected in the participants’ pupillary responses when viewing the paintings for the first time. The use of pupillometry for hedonic research is based on the “arousal potential” account developed within empirical esthetics (Berlyne, 1971; Martindale, 1990). It is also inspired by the circumplex model of affect (Russell & Barrett, 1999; Kuppens et al., 2013), in which “arousal” is one of the two core dimensions of all affective responses (the other being “valence”). The pupil response has been shown to be a valid and reliable index of arousal, also at the cognitive level (Kahneman & Beatty, 1966; Laeng & Alnæs, 2019). Indeed, several previous studies have used pupillometry during esthetic judgments, whether of human faces (Blackburn & Schirillo, 2012; Laeng & Falkenberg, 2007; Liao et al., 2021) or sexually attractive bodies (e.g., Hess & Polt, 1964; Rieger & Savin-Williams, 2012) or an appealing designs or art objects: (e.g., Kuchinke et al., 2009; Laeng et al., 2016). In a previous study on color rotations, Taniyama et al. (2023) used pupillometry while participants provided liking ratings of 180° hue-rotated paintings versus their originals, for either paintings of flowers or abstract compositions. They found that the originals were rated higher (especially with flowers), while pupils dilated more for the originals than the 180° rotations. Therefore, using abstract art for the color manipulations used in the present study has the obvious advantage that, after rotations, unnatural colors are avoided altogether for known objects (e.g., flowers, fruits, faces) and scenes (e.g., blue sky). Previous studies (e.g., Vessel & Rubin, 2010) have shown that, in general, there is low inter-observer agreement in preference with abstract images compared to realistic images, which may increase the chance to obtain diverse physiological responses (Vessel et al., 2012).

Remarkably, as mentioned, one pupillometry study by Johnson et al. (2010) showed that, even when subjective reports or ratings indicated that all orientations of a Mondrian were equally liked, the participants’ eye pupils showed greater dilations to the original orientation of the paintings. This finding suggests that pupillometry, like forced choices, may be able to reveal even slight differences in preference that noisy ratings may not be able to reveal. Alternatively, pupillometry may reveal the presence of a feature that captures attention and affects behavior, therefore not necessarily exposing small differences in esthetics. Given that pupillometric measures could be sensitive to slight hedonic difference or unconscious biases (Laeng et al., 2012), it is relevant to add this psychophysiological method to the toolkit of research on hedonic responses.

### Method

#### Participants

Power analyses were based on a previous study from our lab on esthetic preferences in the design of wine labels (Laeng et al., 2016), where participants chose the preferred “bottle” among four images that showed variants of a same label type as well as rating each label alone, while pupil responses were registered. In that study, we found effect sizes ranging from d = 0.47–0.52. Hence, a power analysis established that a sample size of at least *N* = 30 would be appropriate for the present experiment, given its similarity in measurements of hedonic values. Thus, we recruited thirty-one volunteer participants (24 females; mean age = 25.2 years; SD = 3.04) among students at the University of Oslo. They had normal or corrected-to-normal vision and were all art “novices,” meaning that none of them had any formal artistic education (mean score = 7.09; SD = 3.2 on the Art Experience Questionnaire; Chatterjee et al., 2010). The “content” of a painting seems to be the determining factor for art novices (Leder et al., 2004) and artistically experienced people enjoy art in a significantly different way than art novices (Augustin & Leder, 2006; Bimler et al., 2019; Cupchik & László, 1992). All participants signed an informed consent form before participating in the study.

#### Stimuli and Apparatus

Stimuli consisted of the 22 original abstract art paintings used in Experiment 1. The software used for generating the hue rotations was MATLAB 2018b (Mathworks, Natick, MA). Hue rotations were spaced at 90° intervals on the a*b* plane of the CIELAB color space, creating three different versions of each image from the original (or 0°): 90° rotated, 180° rotated, and 270° rotated. All had the same mean chromacity as the original, but very different hues. Because each painting's L* is identical for all rotations, there were no changes in RMS contrast (M = 0.193, SD = 0.048) based on luminance. None of our participants were familiar with the specific paintings presented, as confirmed at debriefing. Eye data were recorded by using a binocular, infrared, remote eye-tracking device (SMI RED250). Testing took place in a windowless room in the cognitive laboratory at University of Oslo, with an illuminance of 195 Lux. All images were displayed on a Dell LCD monitor with a display resolution of 1680 × 1050 pixels (color-calibrated using Spyder 4 Elite); the screen being positioned at 70 cm viewing distance from a chinrest. The eye tracker sampled the pupil diameter at a rate of 60 Hz from both eyes. We used SMI's *BeGaze* software for extracting the pupil diameters (in mm). All baseline images were adjusted by averaging pixel levels using Adobe Photoshop so that no change in average luminance levels occurred on screen when seeing soon after the respective target painting.

#### Procedure

At the beginning of the experiment, each participant completed the Farnsworth-Munsell 100 Hue Color Vision Test, to evaluate each participant's visual sensitivity to color, since such sensitivity can be associated with the esthetic experience of the viewer (Spehar et al., 2015). Afterward, participants filled out the Art Experience Questionnaire (Chatterjee et al., 2010). In the laboratory session, participants were seated at 50–60 cm away from the laptop screen and the eye tracking bar. All participants were asked to keep their eyes open when the paintings were shown and refrain from eye blinks. In each trial, an empty gray image was shown as a baseline measurement. The shade of gray of each baseline image was adjusted for luminance (by use of *Adobe Photoshop*) and size to the immediately following test image. Each baseline image appeared for 2,000 ms while each subsequent test image was shown for 10,000 ms. This laboratory session consisted of two blocks: first, a *“passive viewing”* condition; second, an “*active viewing”* condition, where the same sequence of images was presented again but this time the participants rated, by clicking with the mouse on the chosen level of the scale, for “liking” each test image on a 7-point Likert scale ranging from 1 (*dislike very much*) to 7 (*like very much*), with 4 as the neutral point (*neither like nor dislike*). In other words, the difference between the passive and active conditions was simply in the instructions at the beginning of the block, where in one case participants simply looked at the images, whereas in the other case, they had to look and think about a rating for the painting’s esthetic appeal, reporting this right after its presentation. The picture stimuli were presented full screen to the participants using *Experiment Center* by SMI, which also recorded pupil diameters with the eye tracker. All instructions appeared on the computer screen.

Two months after completing the laboratory session, all participants were contacted by email which included a PowerPoint presentation with a total of 22 slides, each presenting all four versions (original, 90° rotated, 180° rotated, and 270° rotated) of one of the abstract paintings used in the laboratory session. Note that participants were not initially informed about a follow-up test using a forced-choice test of relative preference. The participants were asked to work at their convenience on their personal computers and make a choice of the preferred version of each painting by dragging a black star symbol onto the image they liked the best within each slide and then save the file and mail it back to the experimenter (EAA-P). Twenty participants, out of the original 31, replied with complete answers.

### Results

All data were analyzed with both classic, frequentist, analyses, and Bayesian (F_1_) analyses (Dienes, 2014), by use of JASP software (https://jasp-stats.org/). All ratings were averaged for each hue rotation (0°, 90°, 180°, 270°) and analyses of variance were consequently by-item, using each painting (*N* = 22) as the random factor.

#### Ratings and Pupil Responses

**Figure 6.**
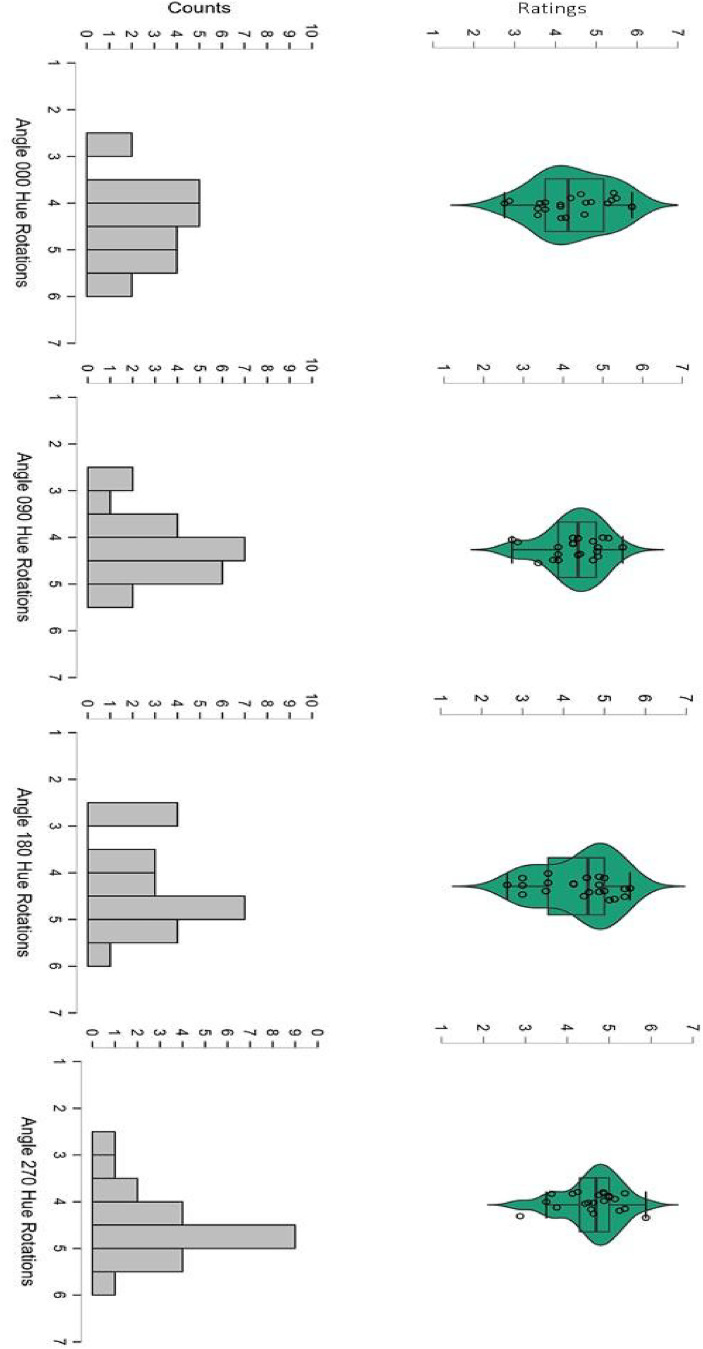
Top row: Violin plots of hedonic ratings of abstract paintings (original: Angle = 000; and hue rotated: Angle = 090, 180, 270). Bottom row: Histograms of rating counts along the scale (1–7).

The histograms and violin plots in Figure 6 show the distributions of ratings in each of the four conditions. The adjacent violin plots suggest that ratings were highly similar for each rotated version, including the original nonrotated paintings, and that all average liking ratings were close to the point of neutrality (i.e., a score of 4). The mean rating for the 0° rotation (or original painting) was 4.42 (SD = 0.89; minimum mean rating = 2.75, maximum mean rating = 5.86); mean rating for the 90° rotation was 4.29 (SD = 0.70; minimum mean rating = 2.71, maximum mean rating = 5.50); for the 180° rotation was 4.38 (SD = .92; minimum mean rating = 2.62, maximum mean rating = 5.61); and for the 270° rotation was 4.60 (SD = 0.70; minimum mean rating = 2.88, maximum mean rating = 5.86). As the histograms of Figure 6 illustrate, neither the maximum value of 7 (“*like very much*”) nor the minimum value of 1 (“*dislike very much*”) were ever chosen for any of the images. The overall pattern gravitated around a mode of esthetic neutrality or hedonic indifference toward the original paintings as well as their transformed images.

A repeated-measure ANOVA on ratings confirmed no statistically significant effect of hue rotations, *F*(3,63) = .779, *p* = .499. A Bayesian repeated-measure ANOVA revealed a F_1_ = 6.736, further supporting the conclusion that none of the four versions were either liked or disliked over any of the other versions. In addition, a separate repeated-measures ANOVA was conducted on the pupillary change data during passive viewing. The mean pupil change for the 0° rotation (or original painting) was −.104 (SD = 0.071); for the 90° rotation = −.075 (SD = 0.075); for the 180° rotation = −.083 (SD = 0.92); for the 270° rotation = −.106 (SD = 0.089).

**Figure 7.**
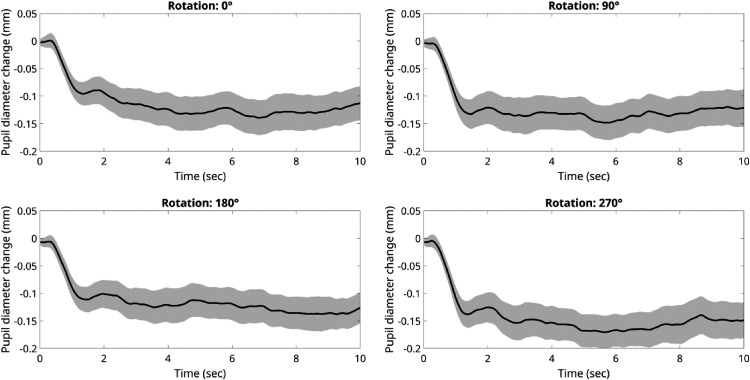
Waveforms of pupillary changes for abstract paintings (original: Angle = 0; and hue-rotated: Angle = 90, 180, 270). The shaded area around the average diameters’ line indicates 95% confidence intervals.

The small and overall negative pupillary changes (Figure 7) indicate the absence of increases in arousal, consistent with a generic indifference toward the artworks, and no differential pupillary changes over time (after an initial constriction from baseline) and across conditions. A Bayesian repeated-measure ANOVA revealed a F_1_ = 7.132, supporting the null hypothesis.

All in all, the pupillometry results concord with the ratings in the active condition, showing no significant differences in pupil diameter evoked by the color rotations, *F*(3,21) = 0.26, *p* = .85. The overall pattern of results is consistent with a lack of arousal potential in the original paintings or of their transformed images, at least for our sample of novices.

A simple linear regression was also conducted using the absolute pupil sizes during the active and passive conditions, which showed extremely similar pupil diameters when viewing the images in the two blocks, *F*(1, 86) = 891.81, *p* < .0001, *R²* of .912. Thus, pupil diameter differed minimally between repetitions and whether a rating judgment was requested or not. Interestingly, this very high correlation in the arousal measure argues against the possibility that, given that the active rating session was preceded by the passive viewing, the latter session may have suffered from effects due to habituation, fatigue, or boredom.

#### Forced Choices

**Figure 8.**
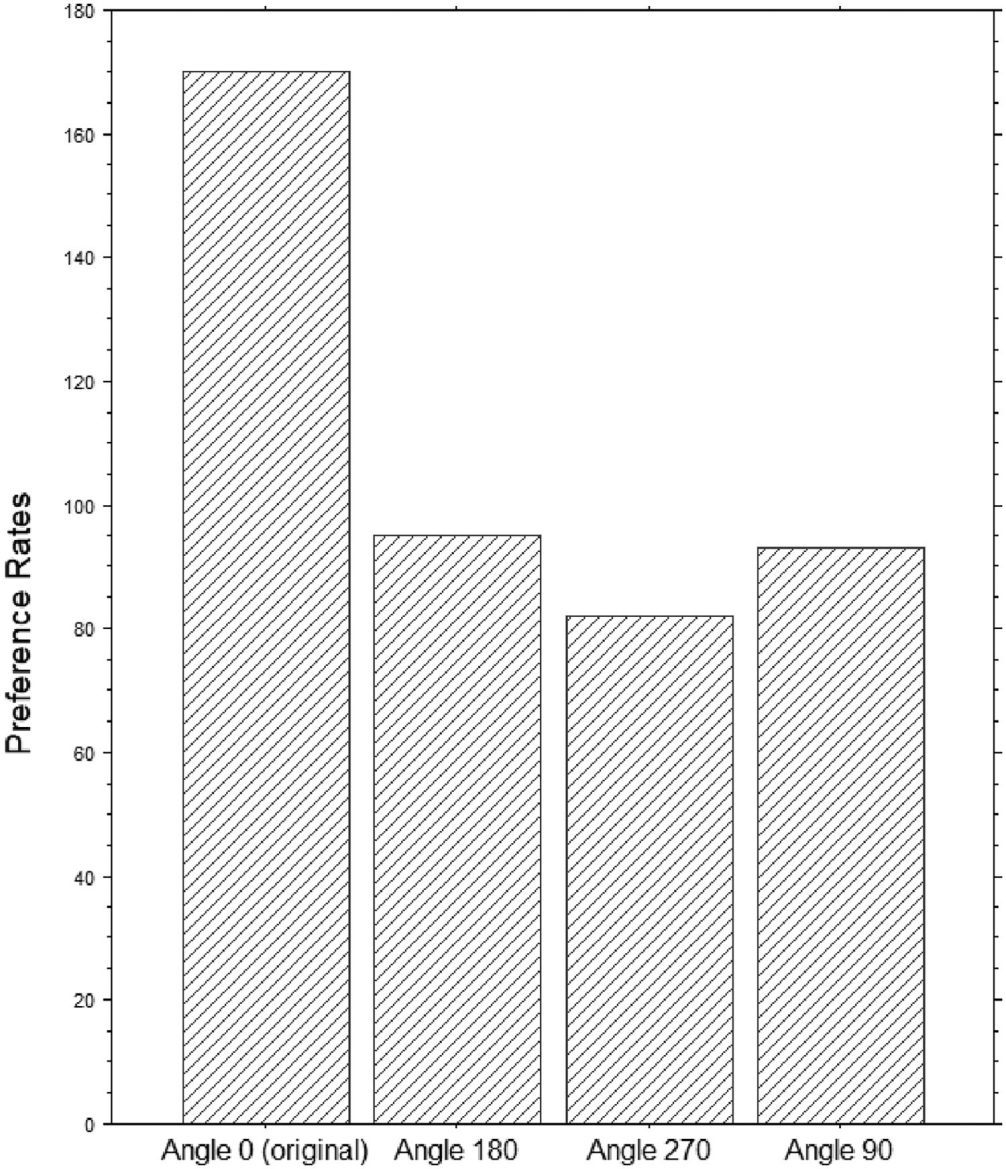
Histogram of rates of preferred image (original: Angle = 0; and rotated: Angle = 90, 180, 270).

A multinomial Chi-square test was applied to the observed rates of choices for a specific version, in the forced-choice test conducted at home with 20 of the original 31 participants. Despite this test was conducted outside the laboratory and in rather uncontrolled conditions (e.g., different computer screens, likely not color-calibrated), we obtained a highly statistically significant effect, *χ2* (3) = 44.53, *p* < .0001. Figure 8 shows that preference for the original version of the paintings over the hue-rotated versions was nearly the double than for any of the hue-rotated versions, replicating findings from previous studies that also used a forced-choice method, either in the laboratory or online.

**Figure 9.**
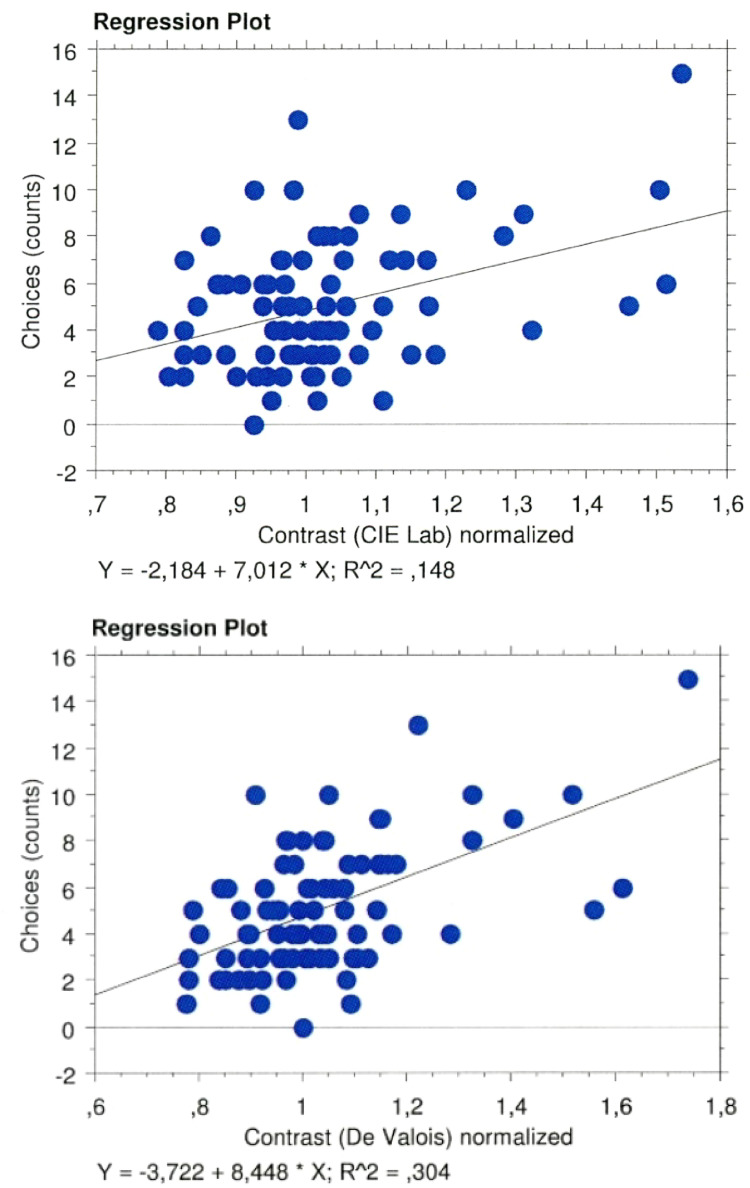
Regression plots of choices over color contrast values within CIELAB (top panel) and De Valois spaces (bottom panel).

To assess the relationship between the color contrast values for each image and choices, we regressed the counts or frequency of choice for a specific angle over the levels of contrast of each painting's rotation; this regression analysis revealed positive (see Figure 9), moderately significant, correlations (CIELAB: *t* = 3.9, *p* = .0002, R^2^ = .148; De Valois: *t* = 6.1, *p* < .0001, R^2^ = .304).

#### FM100 Test

All participants had FM100 scores within norms for their age (as computed with an online score calculator (by Béla Török: https://www.torok.info/colorvision/fm100.htm). Since previous research has shown that visual sensitivity can be associated with the esthetic experience of the viewer (Spehar et al., 2015), we performed a simple linear regression between the average hedonic ratings of each participant and their Farnsworth-Munsell 100 Hue Color Vision Test scores; no significant relation was found, *F*(1, 24) = .107, *p* = .75, *R*² = .004.

### Discussion

We investigated esthetic judgments with two popular methods used by psychologists in measuring esthetic preferences: (a) ratings on an ordinal scale and (b) a forced choice out of multiple items (i.e., four, as in the previous studies). In addition, (c) we monitored pupil diameters while participants were either observing passively each single image or while deciding on a hedonic rating.

We found that when forced to make a choice our novice participants were more likely to choose the original painting compared to another three hue-rotated versions, replicating the results of several studies (e.g., Nascimento et al., 2017) with the same method or stimuli (Nakauchi et al., 2022). However, when asked to estimate the hedonic value (liking) of each image on a 7-step Likert scale varying from “*strong liking*” to “*strong disliking*” and a central neutral point, judgments were on average neutral, and no significant difference emerged between original and any color-rotated images (Figure 6). That the latter is a null finding was confirmed by a Bayesian factor strongly supporting the conclusion (Dienes, 2014) of no esthetic differences across angles. Consistently with that, average pupil diameters did not change across original and color-rotated single images, in both the passive and active conditions, which were also strongly correlated. Hence, based on these hedonic ratings and their pupil responses, it appears that our group of novice participants neither liked nor disliked any version of the paintings despite selecting more often, when forced to make a choice, the original paintings out of three hue-rotated versions. We note that it may take some expertise with the visual arts to fully appreciate abstract artwork (e.g., Augustin & Leder, 2006; Bimler et al., 2019) and we need to stress that our participants were art novices.

As pointed out by several researchers (e.g., Spehar et al., 2015), familiarity with specific color combinations could also bias visual processing. In the present case, a possibility is that a lifetime exposure to specific natural colors (i.e., the “color statistics” of natural scenes) could bias preferences of artists and viewers of visual art toward these natural combinations (Montagner et al., 2016; Nascimento et al., 2021). Nakauchi et al. (2022) showed that recomposing these artificial parts as patchwork images from different paintings led to the original to be typically preferred despite any original color composition would be destroyed. One offered explanation is that the original art objects share very similar regularities with images of nature that possibly is violated by the digital color rotations (Graham & Redies, 2010; Montagner et al., 2016; Taylor et al., 1999, 2011). It is known that art paintings that deviate from natural spatial properties (Fernandez & Wilkins, 2008) are found unpleasant.

However, a study with a large sample of participants (*N* = 31,353) and paintings (1,200 items of different genres; see Nakauchi & Tamura, 2022) failed to find that the preferred paintings were also those that closest resembled the colors of natural scenes (sampled over 1,200 photos). Instead, the gamut of the sampled paintings was generally tilted toward the yellow direction for brighter colors and the blue direction for darker colors, but neither of these tendencies was found in the sampled natural images. Moreover, there was a bias toward highly saturated reds in paintings compared to natural scenes, which raises the question whether this bias might reflect the associated value of certain hues to some relevant natural things (e.g., Palmer & Schloss, 2010; Taylor et al., 2013).

Altmann et al. (2021) also suggested that rotations may introduce adverse effects on color interactions because of differential effects over the perceived category boundaries of colors (cf., Conway, 2012; Fider & Komarova, 2018). Interestingly, they also reported that participants perceived the original color compositions as more colorful or richer than rotated versions. They suggested that color categories that have narrow boundaries (e.g., yellow) will be more strongly disrupted than those with broad perceptual boundaries, and even moderate rotations may leave some colors within the same category while other colors will shift categorically, despite the coordinate distances in CIELAB space among hues remain constant. Note also that rotating hues around the mean of the opponent color axes in CIELAB does not modify the appearance of elements in paintings that are completely black or white (i.e., either unsaturated or achromatic). However, for the artists and viewers these may be essential elements of their color palette (of pigments), just like with the chromatic hues; thus, another unexplored possibility is that these achromatic elements interact in a relevant or salient manner with the original hues and preferences may not be exclusively driven by interactions between the chromatic colors.

## Experiment 3: Effects on Forced Choices, in an Online Experiment, of Lowering or Enhancing Color Contrast of Same Artworks

One should seriously consider the possibility that color contrast per se contributes to a painting's esthetic value and that achieving strong color contrast is what some painters aim for when they combine colors. Indeed, the previously mentioned analyses of the paintings by van Gogh, showing abundant use of complementary colors (Berezhnoy et al., 2007), may provide an example of a celebrated painter attempting to achieve just that effect. In other words, this idea considers color contrast as a “perceptual primitive” of visual esthetics (cf. McAdams et al., 2023). To directly test the causal role of differences in color contrast on forced choices, we run an online experiment where the same original paintings used in the previous lab experiment and feature analysis where digitally transformed to have either lowered or enhanced color contrast. We selected three different levels of color contrast manipulations: 15%, 30%, and 45% change and then presented the original paired with either the enhanced or lowered version of itself, in a 2-alternative forced-choice test, where observers were asked to mouse-click over the version they liked best. Based on our hypothesis that color contrast is relevant for choice, we predicted that when the original was paired to an enhanced version in contrast, it would be now rejected; thus, reversing the usual finding of choosing systematically the original painting of all previous studies. Similarly, we predicted that when the original was paired to a lowered version in contrast, it would win out, just like in all previous studies using forced-choice tests.

The levels of manipulation of contrast included a 15% change that we suspected to make difficult noticing a change in color contrast, whereas the other two levels would show very noticeable changes. To assess how detectable these levels of contrast were and whether these discriminations were symmetrically accurate, when either lowered or enhanced, we requested the same online participants to complete a second online test, where they judged whether the two paintings, again showed side-by-side, looked the “same” or “different.” Note that we did not specify in which way they could look different, though we only manipulated the color contrast, leaving all the other features of hue or luminance unaffected.

### Method

#### Participants

Participants were recruited via email and tested online by use of the free web platform *Psytoolkit* (Stoet, 2010). Forty-eight participants (mean age = 35.13, SD = 13.92; 27 females and 21 males) completed the forced-choice test on preference. Among these participants, a group of 28 (mean age = 36.07, SD = 13.14; 13 females and 15 males) chose to continue and completed the same/different discrimination test.

#### Stimuli and Apparatus

**Figure 10.**
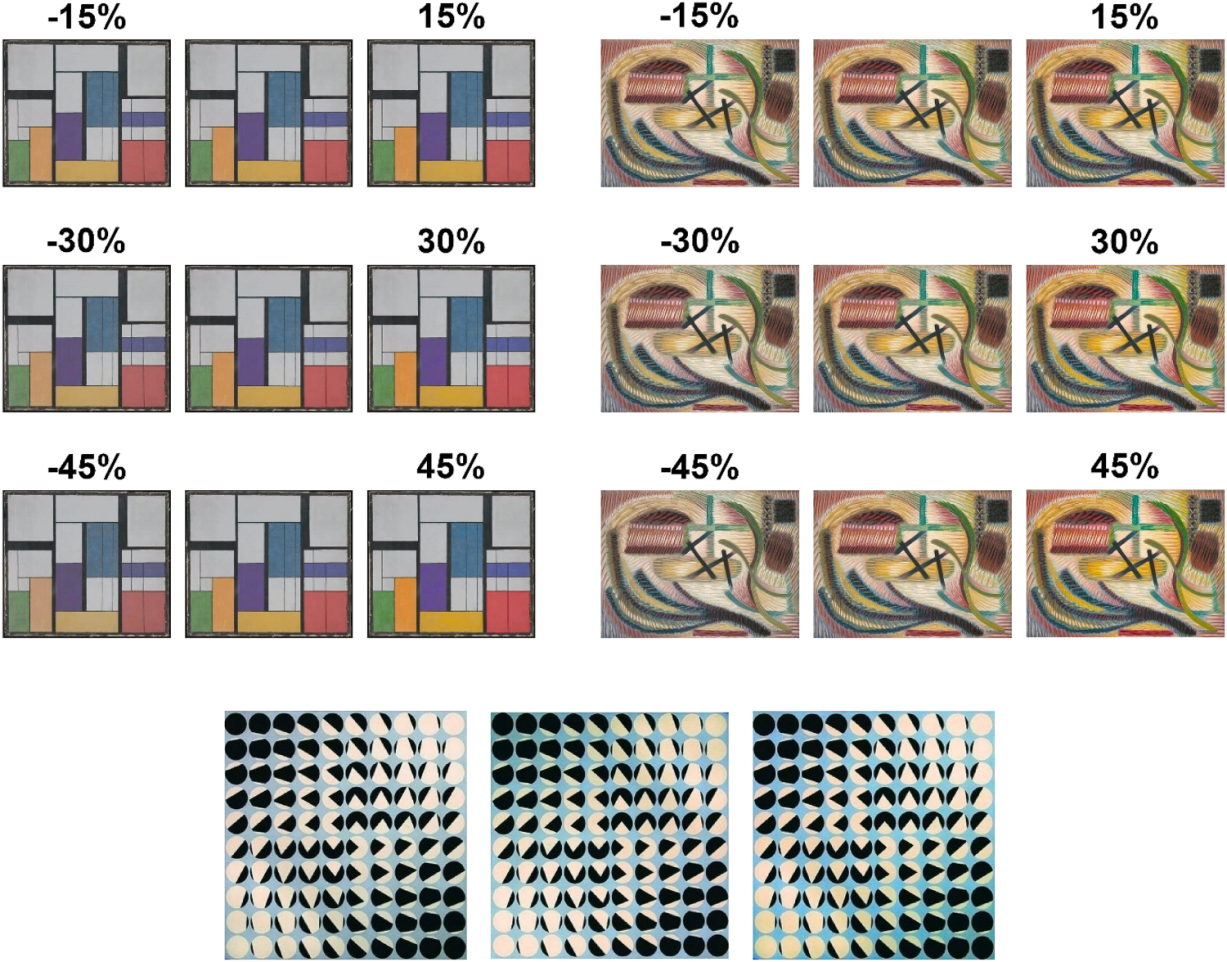
Example of two paintings that were visibly affected by contrast changes (15%, 30% and 45%). The central patterns in both top, left and right, painting's panels show the original painting, repeated to facilitate comparisons, The painting in the bottom row present a painting where the changes in color contrast were minimal (the central pattern is the original and those on the side show, on the left, a decrement of 45% contrast, and on the right an enhancement of 45%).

Stimuli consisted of the 22 original abstract art paintings used earlier in the feature analysis section and six versions with transformed color contrast either lower or higher than the original, in steps of 15%, 30%, and 45% changes (see Figure 10 for examples of the stimuli). Note that color contrast was adjusted by rescaling the saturation values for each hue (divided into 1,000 unique hues) through compressing or expanding the saturation range by 15%, 30%, or 45%, while keeping the minimum saturation level of all hues at the original. Points falling outside the sRGB gamut after contrast adjustment were set to the surface boundary of the sRGB gamut projected in CIELAB space, by reducing saturation while keeping the hue angle. The contrast adjustment procedure caused no changes in hue.

All participants were asked to perform the tests by using the color screen of a personal computer. The use of smartphones or tablets was prevented by PsyToolkit. We also recorded information through the participants’ browser about the computer screens’ sizes used by each participant.

#### Procedure

At the beginning of the experiment, each participant filled out an online version of the Art Experience Questionnaire (Chatterjee et al., 2010) and completed an online version of the Ishihara color test to evaluate each participant's visual sensitivity to color, both presented in *PsyToolkit* before the preference test began. Plates 2–13 of the Ishihara 24 plate test were shown centrally on screen with the question “What number do you see?” along with three possible answers (the correct number, an incorrect number, or the text “No number”).

For the forced choice preference task, participants were randomly assigned to one of the contrast-adjusted versions. Each of the 22 original abstract art paintings were paired with the contrast adjusted version of the same painting, one version with increased contrast and one version with decreased contrast, creating 44 pairs to be evaluated by the participant in a total of 44 trials The original and adjusted painting was presented side by side centrally on the screen (black background) separated by 100 pixels. For each pair, the left/right position of the paintings was determined randomly. All 44 pairs were presented in random order for all participants. Before each trial there was a central fixation cross for 1,000–1,500 ms (randomly set).

For the discrimination task, all participants were presented with pairs consisting of the original painting and a contrast adjusted version of the painting (all three contrast adjustments were used in this test), in addition to 22 pairs consisting of the original painting both to the left and right. This gives a total of 88 trials. For each trial, the left/right position, and whether the adjusted member was increased or decreased in contrast was determined randomly. Each pair was shown for 5,000 ms followed by a blank screen for 300 ms after which the mouse cursor and the words “Identical” and “Different” appeared centrally on screen. Participants used the mouse to indicate whether they thought the two paintings were identical or different. All 88 pairs were presented in random order for all participants. Before each trial there was a central fixation cross for 1,000–1,500 ms (randomly set).

### Results

*Forced choices*. The number of choices for either the original or the transformed image were counted in each condition with different percentage of change in color contrast (15%, 30%, and 45%). These frequencies were then analyzed with separate Chi-square analyses, using *Statview* software, for each percentage condition as well as for the whole data set ignoring conditions. The online Ishihara test identified one participant with low scores; however, excluding this participant did not change the results and we present here results from the full sample.

**Figure 11.**
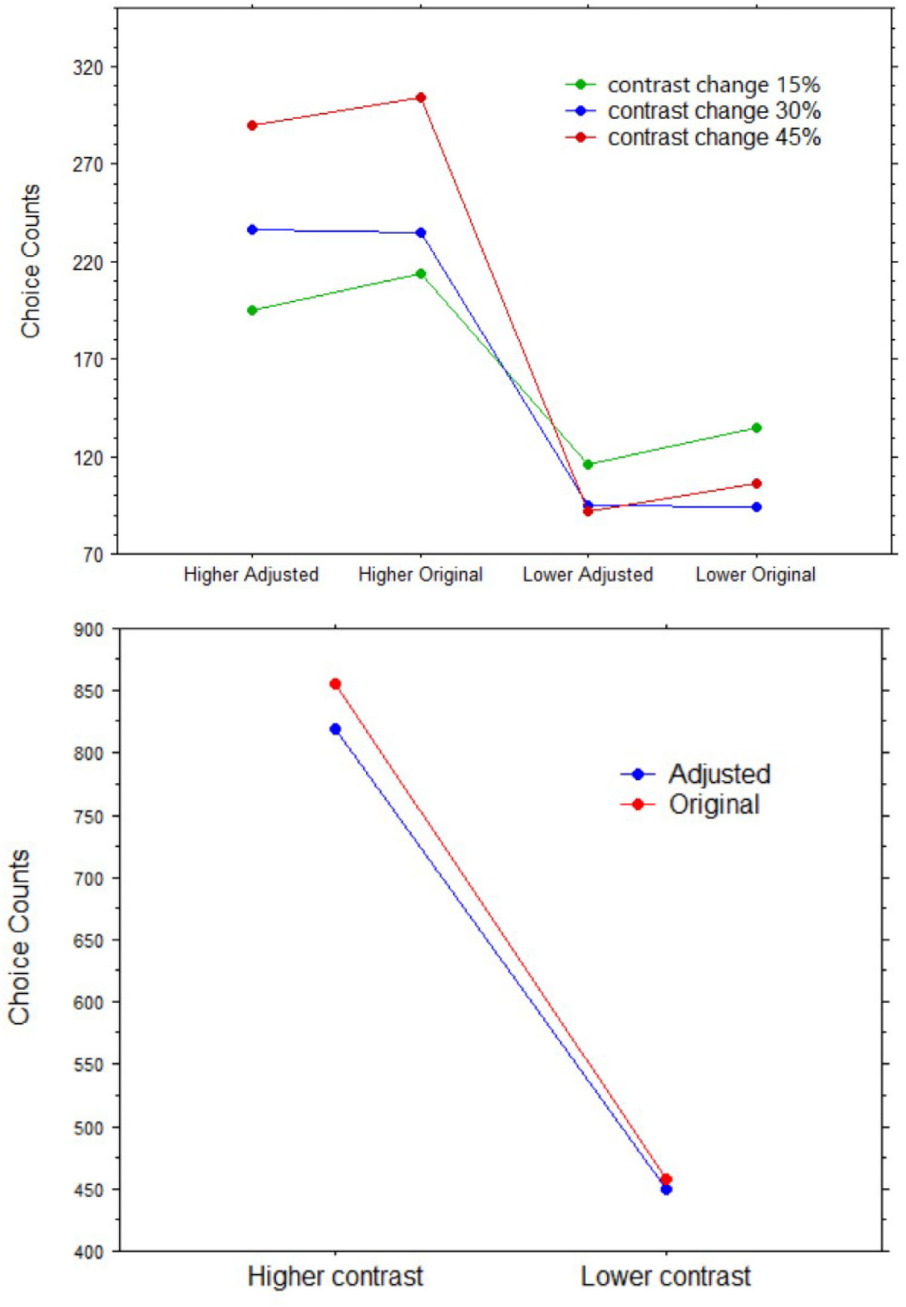
Top panel: Choice counts of images (original vs. adjusted) with different percentages of color contrast adjustment (15%, 30%, and 45%), split by direction of change (lower or higher color contrast). Bottom panel: Choice counts of images (original vs. adjusted) split by direction of change (lower or higher color contrast; both 15% change).

Figure 11 shows frequencies of choices in each of the percentage conditions as well as when grouped together for all participants in Table 1. Clearly, for all percentage changes in color contrast, considered separately or all together, our predictions were confirmed. When the original was paired to an enhanced version in contrast, choices were more frequent for the latter than the former; that is, we observed a complete reversal of the standard preference for the original painting. Similarly, when the original was paired to a lowered version in contrast, regardless of the percentage of change, it stood out in perceptual salience, and consequently, the original was preferred in the forced-choice test like in all previous studies (where color contrast was highly likely to be lower).

**Table 1.** Contingency table of frequencies in which an image in a pair (adjusted or original) was chosen, split by direction of 15% change in color contrast (higher, lower).

	Adjusted image	Original image	Totals
Higher color contrast	819	458	1,277
Lower color contrast	450	855	1,305
Totals	1,269	1,313	2,582

All the Chi-square values obtained in the contingency tables yielded highly significant results. For the 15% change condition: Chi-square = 37.95, Phi = .24, *p* < .0001. For the 30% change condition: Chi-square = 120.49, Phi = .43, *p* < .0001. For the 45% change condition: Chi-square = 198.25, Phi = .50, *p* < .0001. For all conditions together: Chi-square = 146.78, Phi = .33, *p* < .0001. Note that the Phi values (also known as Cramer's V) are equivalent to the correlation coefficient *r* or to a measure of effect size in statistical power calculations, increased from the lowest 15% adjusted images to the 45% images.

*Discriminations*. Changes in the preference for the higher color contrast within a pair were dependent on how visible the enhancement, or decrement, in contrast was, as confirmed by the analyses below on the discriminability between original and adjusted images. We first computed the proportions of trials in which each participant was accurate in detecting whether the image pair showed identical images (i.e., responded “yes”) or not (i.e., responded “no”). All trials with identical images were correctly identified (i.e., there were no misses). Instead, the proportions in which different images were detected varied across percentages of adjustments.

**Figure 12.**
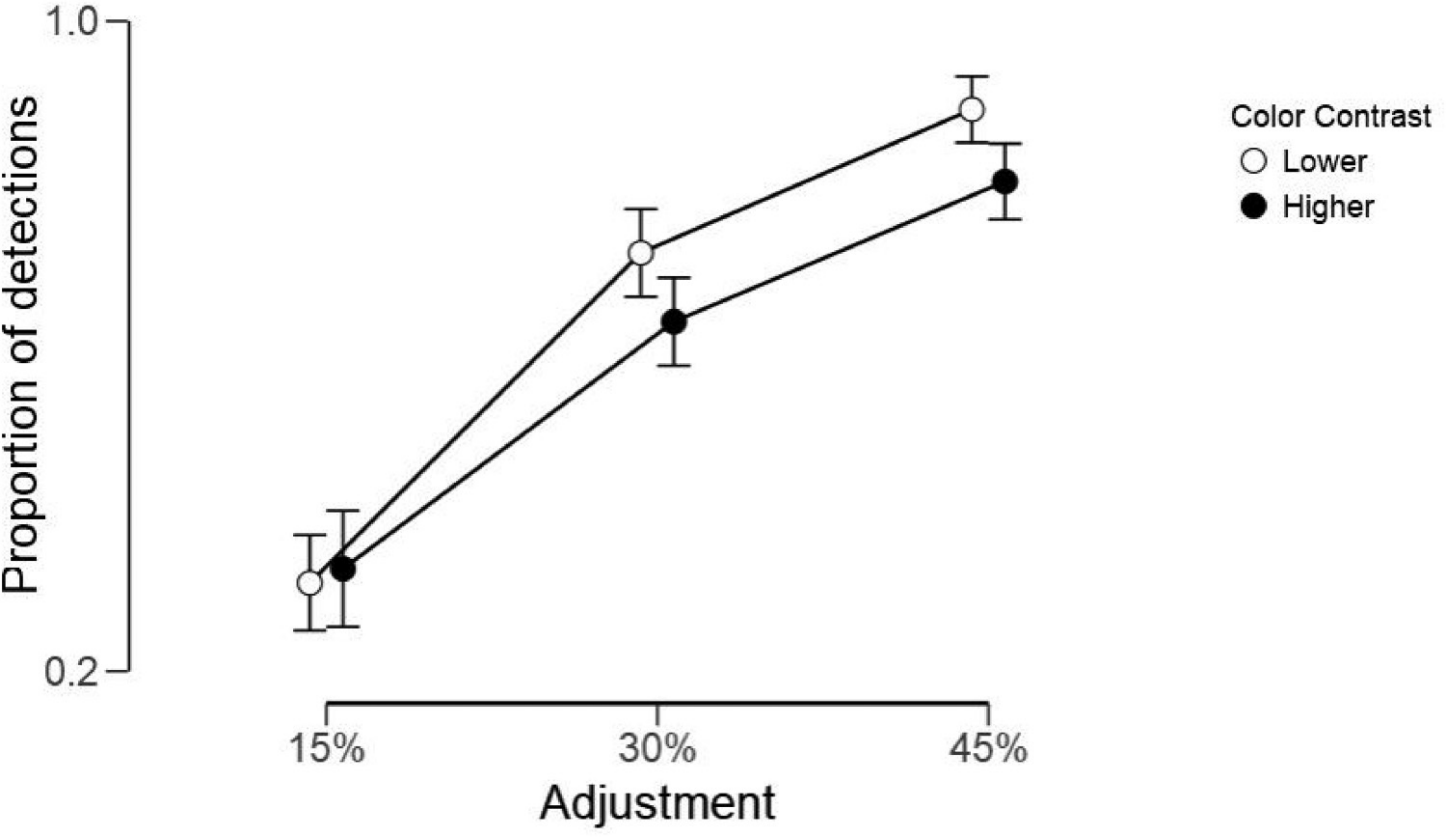
Proportions of correct detection that the images (original vs. adjusted) were different for each percentage of color contrast adjustment and split by direction of change (lower or higher color contrast).

Hence, we performed a repeated-measures ANOVA, using *JASP* software, using correct detections of a difference, with Color Contrast (higher, lower) and % Adjustment (15, 30, and 45) as the within-subject factors. A preliminary ANOVA also included as a between-subjects factor the Screen Size (small, large, defined by a median split of the range of screen sizes), but there was no significant main effect or interactive effects, hence this factor was removed from the analysis. We found significant effects for both Color Contrast, *F*(1, 27) = 10.03, *p* = .004, η² = .27, and % Adjustment, *F*(2, 54) = 179.12, *p* < .0001, η² = .87, but no interactive effect between the two, *F*(2, 54) = 2.29, *p* = .11 (Figure 12 illustrates these results).

Clearly, most differences were noticed for the higher levels of contrast (proportion range: .79–1.0), whereas for the lowest (15%), a difference was noticed on average in only a .31 or .32 proportion (lowered and enhanced contrast, respectively). The lack of an interactive effect suggests that enhancement or decrement of contrasts were likely to be noticed with equal likelihood.

### Discussion

This online experiment revealed that even small differences in color contrast affected how likely were observers to choose an image as preferable. There was a clear tendency to choose the image with relatively higher color contrast, regardless of whether it displayed the original painting (in conditions were the adjusted image had lower contrast than the original) or the transformed image (in conditions were the adjusted image had higher contrast than the original).

We chose to test such percent changes in color contrast since they seemed most likely to be within the range of contrast changes that can occur after hue rotations. However, we did not test above the 45% contrast change, and we can only speculate that higher changes—particularly when enhancing the contrast—could reach a turning point in which the colors’ appearance becomes unnatural and possibly detrimental to esthetic appeal. Note that, according to the logic of previous studies, if we took the results of forced choices at face value as genuine esthetic preferences, we should conclude that by enhancing the color contrasts, at least within the range tested here, we “improved” the artistic quality of the paintings, beyond the intentions and skills of the original painters. However, this is not necessarily our preferred conclusion, as we discuss later in the general discussion.

Consistently with what we had hypothesized, and as seen in previous studies, the original painting was preferred when it was also the item with the highest color contrast. However, the original painting was more likely to be chosen in the forced-choice test only when the alternative, adjusted, image had relatively lower color contrast. Hence, the present findings reveal that color contrast plays an important role for preferences for paintings in a forced choice situation. Moreover, this experiment also revealed that changes in hues, as in the standard rotation experiments, were not necessary for revealing a preference for the original.

Hence, the present results together with our previous experiment with hedonic ratings and pupillometry could suggest that “preferences” might take place at a nonesthetic level, dependent on the perceptual salience of some items among others biasing attention. It is well known that salience is a strong attractor of attention (e.g., Parkhurst et al., 2002) and this asymmetry in attention may decide which image will be selected in a forced-choice situation (Shimojo et al., 2003). Indeed, Isham et al. (2013) showed in a forced-choice task that looking time predicted choice but not necessarily the esthetic value of two objects. Finally, we note that choices for the higher contrast images were found also in the condition in which observers were able to detect differences in color contrast only in one out of three of the trials. This bias increased, as shown by changes in effect size across percentages of adjustments, with increased discriminability of the perceptual difference.

## Experiment 4: Effects on Forced Choices, in a Laboratory Experiment With Eye Tracking, of Lowering or Enhancing Artworks’ Color Contrast

The previous experiment revealed strong effects of color contrast on choices; however, the evidence was based solely on an online version of the task, where there was no control on how the images appeared on different computer screens and several other uncontrolled conditions like distractions, differences of room illumination, and use of uncalibrated screens. Hence, we proceeded to replicate the previous experiment in the eye-tracking laboratory where many conditions can be kept constant. We deemed sufficient as well as most relevant to use the lowest percentage of contrast change, tested earlier, of 15% adjustments.

In addition, the use of eye tracking allows to monitor gaze and compare the frequency and duration of fixations on either the original or the adjusted (lower/higher contrast) image. Some previous studies have used gaze durations as signs of implicit esthetic preferences (e.g., in infants who cannot provide explicitly esthetic judgments, e.g., McAdams et al., 2023). In other words, gaze behavior can provide evidence regarding the ability of esthetic features or salient features to capture an observer's attention. An asymmetry in overt attention may be sufficient for deciding which image will be selected in a forced-choice situation (Shimojo et al., 2003).

Thus, we presented again, in a forced-choice test in the eye tracking laboratory, two images side by side, consisting in one of the original paintings shown in the previous experiments and one of the 15% adjustments in color contrast (either enhanced or reduced). We expected to replicate the findings of the earlier online experiment; that is, choices of the original or of the transformed image would be strongly dependent on whether the color contrast of the adjusted image was 15% lower or higher, respectively, than that of the original.

### Method

#### Participants

Participants (*N* = 30; 19 females; mean age = 27.23, SD = 6.68) were students in psychology at the University of Oslo. In this laboratory session, they were seated at 60 cm away from the screen and the eye tracking camera. All participants were asked to keep their eyes open when each pair of paintings were shown and refrain from eye blinks.

#### Stimuli and Apparatus

We used the same trials and pairs of stimuli with original images and their 15% adjustments in color contrast, as in the online experiment, and with the same apparatus, the color-calibrated screen, and in the same laboratory room that was used in the earlier laboratory experiment, when we collected hedonic ratings of color-rotated images.

#### Procedure

The stimuli were shown for 10 s, after which a question appeared on screen, asking to press a key, on the left or right side of the computer keyboard when the preferred image was also either on the left side of the screen or on the right side.

### Results

#### Forced Choices

The number of choices for either the original or the transformed image were counted, and frequencies were then analyzed with a Chi-square analysis, using *Statview* software. Table 1 shows frequencies of choices for the original versus adjusted (higher or lower contrast) images. We confirmed the findings obtained in the same condition in the earlier, online, experiment. The original was more likely to be rejected when paired to an enhanced version in color contrast or to be selected when it had perceptual salience. The Chi-square test yielded a highly significant result, Chi-square = 227.09, Phi = .29, *p* < .0001. Note that significance was higher in the laboratory than in the online experiment, likely due to the laboratory-controlled conditions. In addition, the effect size was also slightly higher than for the online experiment and for the same percentage of contrast adjustment (Phi = .24) (Table 1).

#### Eye Tracking

We first excluded from each trial data with a latency above 10,000 ms or below 150 ms, and fixations with a dwell time above 3 SD from the mean. We delineated areas of interest (AOIs) with contours tightly corresponding to each painting's image and then computed, from the valid trials, three dependent oculomotor variables: (a) the number of fixations on each image in a pair; (b) the average dwell time on each image in a pair; (c) the average pupil diameters when looking at each image (by defining an area of interest for fixations). In general, within each AOI, gaze tended to be distributed widely, with some crowding at the center of each pattern.

**Figure 13.**
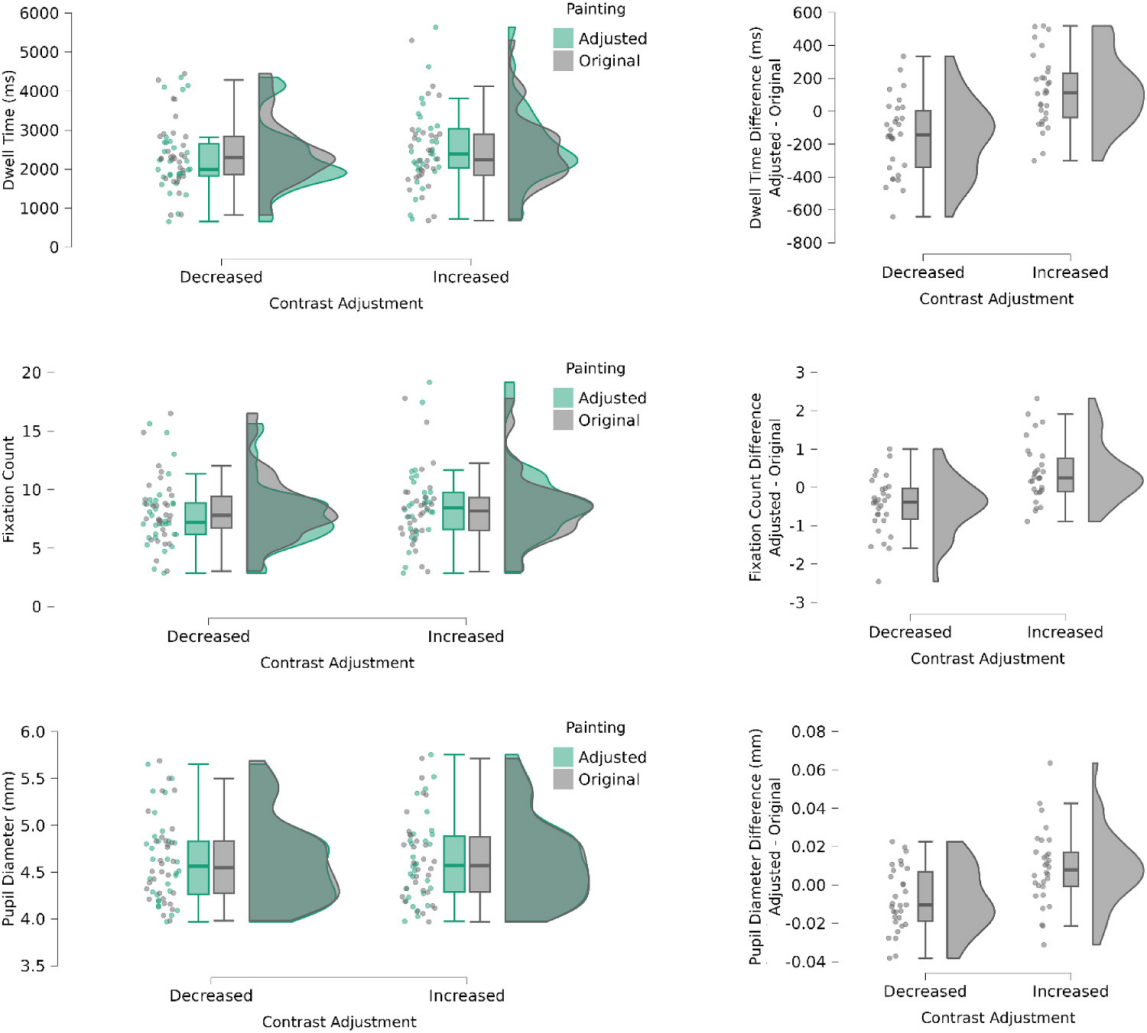
Boxplots of dwell time (left top panel), fixation counts (left middle panel) and pupil diameter (left bottom panel) for images with relatively increased or decreased color contrast (right panels) for its paired original painting. The boxplots in the right column show difference values (adjusted minus original) for each parameter to better visualize the effects of increases versus decreases in color contrast.

We performed two paired, *t*-tests with the above measures comparing the original painting with either the 15% enhanced or reduced color contrast version. We expected that the image in the pair with relatively higher contrast, regardless of whether this was the original painting or transformed image, would receive more overt attention. Indeed, we found that more gaze fixations were directed to higher-contrast image; Enhanced contrast versus Original: *t*(29) = 2.74, *p* = .01 (Cohen's d = 0.5); Decreased contrast versus Original: *t*(29) = 2.74, *p* = .002 (Cohen's d = 0.62). The dwell times on the higher-contrast image were longer than on the relatively lower image; Enhanced contrast versus Original: *t*(29) = 3.1, *p* = .004 (Cohen's d = 0.56); Decreased contrast versus Original: *t*(29) = 3.56, *p* = .001 (Cohen's d = 0.65). The pupil diameter for fixations within the AOI corresponding to the higher-contrast image were larger than for the AOI corresponding to the relatively lower contrast image; Enhanced contrast versus Original: *t*(29) = 2.46, *p* = .02 (Cohen's d = 0.45); Decreased contrast versus Original: *t*(29) = 2.53, *p* = .002 (Cohen's d = 0.46). Figure 13 illustrates these findings for the three measures.

**Figure 14.**
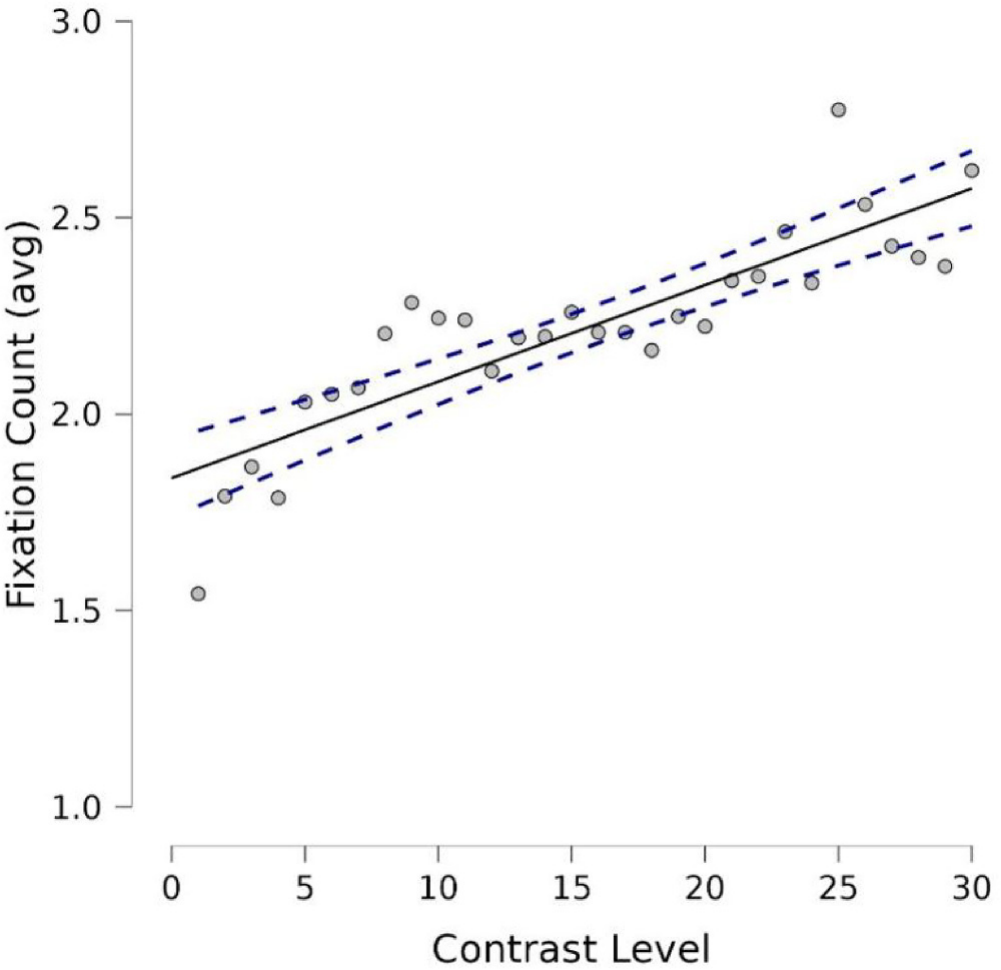
Regression of number of fixations (count) on contrast level (30 steps). Straight line is the interpolation line and dotted lines express the variability.

Finally, to further explore the role of differences in color contrast on the three oculomotor measures, we computed the level of contrast of each image used in the experiment in 30 steps. Specifically, the color contrast map for each painting (each pixel's difference from the average color in the entire painting) was thresholded to 30 levels using quantiles, ensuring that the areas are equal in size (same number of pixels with each contrast level). We then applied three separate Spearman's regression analyses to Contrast Level as the regressor and each of the oculomotor measures as the predicted variables. Only the regression with fixation counts was significant: Spearman's Rho = 0.89, *p* < .001 (see Figure 14); whereas the other two measures missed significance (Dwell time: Spearman's Rho = 0.1, *p* = .6; Pupil diameter: Spearman's Rho = 0.2, *p* < .14).

### Discussion

We expected that esthetic preferences and/or saliency guide attention and therefore predicted that the image in the pair with relatively higher contrast would receive more attention, regardless of whether this was the original painting or a transformed image. Using eye tracking, we found evidence of increased dwell time and frequency of fixations on the image in a pair with 15% more color contrast. Participants also had on average, larger eye pupils, when looking at the higher-contrast image. The findings are consistent with the relatively more salient image in contrast capturing attention. As we mentioned earlier, pupil dilation has been shown to be a generally valid and reliable index of arousal (Kahneman & Beatty, 1966; Laeng & Alnæs, 2019) and Johnson et al. (2010) showed that individuals looking at different orientations of Mondrian paintings had greater pupil dilations to the original orientation. These authors suggested that pupillometry was able to reveal slight differences in preference that noisy ratings were not able to reveal. Interestingly, contrast levels did not predict pupil diameters, which suggest that the pupil did not simply dilate in proportion to contrast but in relation to the relatively salient image in each trial. Finally, we observed a nearly perfect correlation between the pupil sizes during passive viewing and during active ratings, which makes unlikely that the lack of arousal potential was due to a “response compression” toward the central values, which is an artifact that can happen with hedonic scales (e.g., Cardello et al., 2008). Similarly, the pupils’ null effects are unlikely to result from equally evoked opposite valences with equally intense arousal (Janisse, 1974), that is, falling into the so-called “isohedonic trap” (Martindale, 1984). Instead, using Bayesian statistics (Dienes, 2014), we found strong support for no difference in esthetic value or arousal potential for our participants; hence the null finding cannot be attributed to a lack of statistical power.

## General Discussion

The present study gives additional evidence to the robust finding that transformed colors in paintings through rotations within CIELAB space are consistently rejected in forced-choice situations. However, the present experiments also show that when the same viewers can estimate or rate the hedonic value of each painting independently, the original paintings might not be necessarily judged as having differential esthetic value. Based on both the present image analyses, as well as the behavioral and physiological findings of several experiments that we conducted (online or in the lab), we conclude that digital rotations within color spaces introduce some, typically, adverse changes in perceptual saliency. That is, color contrast is weakened by the rotation technique typically used in this line of research and, therefore, the original paintings are likely to stand out as perceptually richer among their variations. Hence, we suggest that in these cases participants will be biased to choose the odd-man-out while the original will be rejected in favor of the contrast enhanced versions.

We first offered a detailed analysis of changes in color contrast after rotation in CIELAB space as well as within an alternative, “physiological” space that we based on the well-known multistage model of color vision by De Valois and De Valois (1993). Not only did we find evidence of significant reduction of color contrast after rotations in color spaces (albeit stronger in “physiological” space than CIELAB), compared to the original paintings, but we also found that contrast predicted the frequency of participants’ choices in the forced situation. In our opinion, all this constitutes strong evidence for the robust “preferences” for the original paintings as mainly reflecting their changes in color contrast compared to any of the rotated versions.

### Can Color Contrast Be Considered an “Esthetic Primitive”?

Using the eye-tracking method, we found that individuals looked longer to the relatively higher-contrast (or salient) image in a pair than the one with lower color contrast. In one of our experiments, this happened independently of whether the higher-contrast picture was the original painting or a version with either 15% enhanced or reduced contrast. Moreover, in forced choice tests, participants tended to choose the preferentially looked images. Such findings are consistent with previous evidence that stimuli with higher esthetic value are looked longer than those with lower values, whether these are design objects (Laeng et al., 2016) or human faces varying in attractiveness (Chelnokova et al., 2014). Developmental studies of “preferential looking” with infants have identified “visual preferences” for several sensory features (e.g., luminance contrast between edges, vertical symmetry, complexity, proportion, contour, brightness, curved contours; Juricevic et al., 2010). As mentioned by McAdams et al. (2023), some of the esthetic responses in adults to visual images could originate from these simple sensory visual preferences observed at the early preverbal stages. For example, infants look longer at attractive faces, as rated by adults, than the less attractive; Damon et al., 2017).

In their own study of infants who were shown visual art works (e.g., landscape pictures, including Van Gogh's), McAdams et al. (2023) found that a combination of chromatic and spatial image statistics accounted for two-thirds of the variance in infant looking, and their looking preferences converged with the adult ratings. Specifically, they identified the amount of variation in the luminance and color saturation of the photo pixels as modulators of both the looking preferences and adults’ ratings and also suggested that these early sensory biases in infancy may potentially be two “perceptual primitives” of esthetics. In relation to our present study, we could suggest that “color contrast” could be added to this growing list of potential perceptual primitives. Future studies may assess whether a color contrast preference is present also in infancy.

Considering color contrast as an esthetic primitive may also accommodate the earlier surprising findings that, when original art that was chopped-up and even mashed-up from different original paintings into novel patchworks, the obtained images were consistently preferred in forced-choice tests over analogous patchworks made from the hue-rotated images. Incidentally, such a finding strongly excludes the idea that the effect originates from the artist's deep intuition of what constitute an optimal or harmonious combination of colors, since these meshed stimuli cannot be considered any longer as “authentic” artworks. Instead, it seems likely that the higher color contrast within the fragments gets inherited in the obtained. This seems a straightforward account for a preference for the meshed-up versions (Nascimento et al., 2021), that would seem difficult to explain otherwise (Nakauchi & Tamura, 2022). Future analyses may throw light on such a possibility.

*Is it possible to show a “preference” while being esthetically indifferent?* As a closing, we would like to present an alternative and cautionary view, that does not assume that such systematic “choices” in forced response tests take place necessarily at the esthetic level (i.e., based on an item's appeal). Namely, by reducing the perceptual salience of the other items in the forced choice set, the original's relatively higher contrast could bias attention toward this item. This attentional capture in turn would increase the likelihood that observers would select it, when “forced” to make a choice, since in this paradigm participants cannot opt out or declare that no specific item is more appealing than the others; in fact, they are forced to give a response, occasionally on the basis of an arbitrary sensory or motor bias.

Thus, we surmise that a systematic choice behavior could happen even in situations where all the viewed images in the set have equal esthetic or hedonic value for most observers. To exemplify this alternative account's explanatory force is necessary to refer to a seminal article by Nisbett and Wilson (1977). They found that individuals can make esthetic-like choices while largely unaware of the existence of nonesthetic properties that strongly influence their choices, albeit in an implicit way. In one of the Nisbett and Wilson's experiments, passerby individuals in a clothing shop were invited to evaluate identical pairs of stockings and select the one with “best quality.” Although the stockings positioned on a right-most tray were typically preferred, none of the participants (*N* = 52) ever mentioned “position” when queried about the reasons for their choices. Note that it is evident that “being rightmost” cannot be proposed to be an underlying esthetic property or primitive but it can only be described as a nonesthetic, sensory-motor, bias.

In fact, our measurements of hedonic values - with both subjective ratings or the objective pupil responses - suggested that our participants did not have any systematic preference, or showed differences, for the hedonic values of the four images. Thus, it seems cautious as well as consistent with all the evidence that we collected (i.e., forced choices, ratings, pupil dilations) to conclude that the original paintings had no differential appeal compared to any of their hue-rotated versions. By considering in parallel all pieces of evidence, without ignoring the presence of contradictory findings between subjective ratings and pupil responses compared to forced choices, the present findings could be consistent with a general process of attentional capture (Parkhurst et al., 2002). Indeed, we found that the images with relatively higher color contrast captured both selective overt attention and stronger intensive attention (cf. Kahneman, 1973), as shown respectively by dwell times and number of fixations versus the pupil diameters. As McAdams et al. (2023, p. 2) pointed out, an observer could be looking more at a particular stimulus for a variety of reasons, such as visibility, complexity, novelty, and familiarity (Houston-Price & Nakai, 2004), or because of low-level perceptual biases that may be innate, or mature very early (e.g., Bornstein et al., 1981). Interestingly, also McAdams et al. expressed caution in concluding that these infants’ visual preferences should be considered “esthetic.”

Hence, we should be cautious in concluding that these color contrast effects are the straightforward expression of differential hedonic experiences. The literature on choice bias warns also of a phenomenon called “choice blindness” (Johansson et al., 2005), where attributions of intentionality (e.g., why do we like someone's face?) might often be misjudged—retrospectively—from watching one's own actions, or choices, without awareness of the real reasons (Eagleman, 2021). Hence, even entirely arbitrary choices can afterward induce a sense of a conscious esthetic choice (i.e., a “choice-induced preference change”; Nakamura et al., 2013). Finally, our findings with gaze's dwell time are consistent with the phenomenon that looking longer at something can influence choice (Isham et al. 2013; Shimojo et al., 2003) and, considering that our measures of pupil diameters showed no differential “arousal potential” of hue rotations, coupled with no differences in hedonic ratings by the same participants, all the evidence converge in suggesting that a forced choice bias (“preference”) could be expressed behaviorally even in the presence of (esthetic) *indifference* between the items.

An objection to the above alternative account is that forced choices may always have greater sensitivity than the rating method in revealing hedonic differences (cf., Gur et al., 1997). Indeed, this assumption seems common in previous studies on the effects of color rotations. Similarly, one could object that the null effect with average hedonic ratings reflected the well-known tendency by observers to select values at the center of the scale, and that may be then ignored as an artifact. Surely, these limitations of scales are generally true; however, to dismiss null findings in ratings ignores the fact that they represent a direct measure of hedonic value. We also observed, in Experiment 2, that the hedonic ratings were equally variable across rotations, and the ratings spanned a large range of the seven-step scale, as visible in the violin plots of Figure 6, arguing against a strong tendency by participants to be biased in selecting the central values. In fact, one previous study that used ratings (Altmann et al., 2021) did find differences using such a scale, which would speak against the idea of a general inability of ratings to probe even slight hedonic differences. Specifically, their group (*N* = 20) of participants showed a disliking for all rotations. It is also of note that their scale had six steps and did not include an explicit indifference (zero) point, so that it should be considered more like a “forced-opinion scale” (de Rezende & de Medeiros, 2022), since the absence of a neutral point force the respondents to choose either a positive or negative opinion. In sum, Altmann et al.’s study appear to confirm the ability of ratings to reveal differences in hedonic judgments of color-rotated abstract paintings, in this case at least as different degrees of displeasure.

## Conclusions

Computerized techniques where the “color volume” of a painting can be “rotated” rigidly around the mean of the opponent color axes in CIELAB space may be able, to some extent, to keep constant lightness and saturation while systematically changing the hues in the images. However, there are good reasons to believe that other perceptual features of color suffer from the procedure, so that the original emerges as the most salient perceptual version of a painting. We showed empirically that color contrast is weakened after rotation in two color spaces and that a group of art novices did not experience much esthetic pleasure or arousal with these abstract paintings; nevertheless, they selected the image with the original colors when forced to choose between these versus those with changed hues. Importantly, we were also able to invert this bias in choice by enhancing the color contrast of the original painting (up to 45%), even in difficult conditions for detecting a perceptual difference (i.e., 15%). The whole evidence accrued here does not support the intuition that an original artwork necessarily yields the most harmonious constellation of sensory attributes. Instead, we suggest here that (a) color contrast might constitute an esthetic primitive, previously ignored in studies of hue rotations; or (b) alternatively, there may be nothing especially “esthetic” about choosing the original artwork in forced-choice tests, whenever—within a set of equally appealing items—some sensory feature is likely to capture attention.
